# A pathogen-specific sRNA influences enterohemorrhagic *Escherichia coli* fitness and virulence in part by direct interaction with the transcript encoding the ethanolamine utilization regulatory factor EutR

**DOI:** 10.1093/nar/gkab863

**Published:** 2021-09-30

**Authors:** Amber B Sauder, Melissa M Kendall

**Affiliations:** Department of Microbiology, Immunology and Cancer Biology, University of Virginia School of Medicine, Charlottesville, VA 22908, USA; Department of Microbiology, Immunology and Cancer Biology, University of Virginia School of Medicine, Charlottesville, VA 22908, USA

## Abstract

Enterohemorrhagic *Escherichia coli* (EHEC) O157:H7 relies on sRNAs to coordinate expression of metabolic and virulence factors to colonize the host. Here, we focus on the sRNA, named MavR (metabolism and virulence regulator), that is conserved among pathogenic *Enterobacteriaceae*. MavR is constitutively expressed under *in vitro* conditions that promote EHEC virulence gene expression. Using MS2-affinity purification coupled with RNA sequencing, the *eutR* transcript was identified as a putative target of MavR. EutR is a transcription factor that promotes expression of genes required for ethanolamine metabolism as well as virulence factors important for host colonization. MavR binds to the *eutR* coding sequence to protect the *eutR* transcript from RNase E-mediated degradation. Ultimately, MavR promotes EutR expression and in turn ethanolamine utilization and ethanolamine-dependent growth. RNAseq analyses revealed that MavR also affected expression of genes important for other metabolic pathways, motility, oxidative stress and attaching and effacing lesion formation, which contribute to EHEC colonization of the gastrointestinal tract. In support of the idea that MavR-dependent gene expression affects fitness during infection, deletion of *mavR r*esulted in significant (∼10- to 100-fold) attenuation in colonization of the mammalian intestine. Altogether, these studies reveal an important, extensive, and robust phenotype for a bacterial sRNA in host-pathogen interactions.

## INTRODUCTION

To colonize a host, enteric pathogens must overcome a variety of hurdles, including competing for nutrients with the microbiota, coordinating expression of virulence traits, and evading host defenses. Enterohemorrhagic *Escherichia coli* O157:H7 (EHEC) is a preeminent example of a pathogen that precisely adapts to its target environment of the colon, with as few as 10–100 bacteria able to cause infection (1). To do this, EHEC exploits a variety of metabolites to sidestep nutritional competition (2) and then traverses the mucus layer to establish a niche at the relatively sterile epithelial border. At the epithelium, EHEC expresses a type three secretion system (T3SS) and effectors which results in intimate adherence to colonocytes and the formation of attaching and effacing (AE) lesions (3,4). AE lesions are characterized by the effacement of the microvilli and rearrangement of underlying host cytoskeleton resulting in the production of a pedestal-like structure beneath the bacterium (5). The locus of enterocyte effacement (LEE) pathogenicity island encodes the T3SS and most of the effectors required for AE lesion formation and is required for host colonization and overall pathogenesis (6–12).

EHEC coordinates expression of traits important for host colonization by sensing signals within the gastrointestinal (GI) tract and precisely controlling gene expression (13). To date, transcriptional regulation of EHEC gene expression is well-recognized to play a key role in niche adaptation (e.g. (14–17)). However, it is becoming increasingly appreciated that post-transcriptional regulation is a critical mechanism for EHEC to control expression of virulence traits (18,19). sRNAs are robust regulators that mediate post-transcriptional gene expression typically by base pairing to target mRNAs and repressing or promoting gene expression (20–22). In EHEC, sRNAs directly affect expression of transcripts encoding T3SS components (e.g., (23)). Moreover, sRNAs may be integrated into transcriptional networks by targeting transcription factors (24,25). To date, most knowledge of sRNAs in EHEC is derived from studies that were performed using nonpathogenic *E. coli* as the model organism. These studies have provided invaluable insights in understanding mechanisms of post-transcriptional regulation in the *Enterobacteriaceae*. Notably, during its evolution, EHEC acquired ∼1.34 Mb of unique DNA (26,27). Besides encoding canonical virulence traits, these pathogenicity islands harbor regulatory sRNAs that influence expression of core genes common to nonpathogenic *E. coli* and EHEC as well as EHEC-specific genes (19,24,28–30). However, the regulatory mechanisms and physiological importance of the majority of EHEC-specific sRNAs remains elusive.

The sRNA sRNA56 was originally identified by RNAseq as present in EHEC but absent in *E. coli* K-12 (28). Another study confirmed sRNA56 expression and location in an EHEC specific pathogenicity-island (19). Overexpression of sRNA56 influences expression of a T3SS apparatus protein (28), indicating this sRNA affects EHEC virulence. Here, we undertook a comprehensive analysis to characterize the sRNA56 regulon as well as the physiological importance to EHEC fitness and virulence. Based on our findings, we propose that sRNA56 be renamed MavR (metabolism and virulence regulator) and use this nomenclature throughout this paper. Using MS2-affinity purification coupled to RNA sequencing (MAPS) (31,32), we identified transcripts encoding several transcriptional regulators as putative MavR targets. Specifically, the *eutR* transcript was significantly enriched. EutR is the transcription factor that activates expression of genes required for ethanolamine (EA) metabolism in the *Enterobacteriaceae*. We provide a detailed mechanism in which MavR promotes EutR expression and EA utilization by stabilizing the *eutR* transcript. Notably, our findings indicate that MavR interacts with the coding sequence (CDS) of the *eutR* transcript to antagonize RNase E-mediated degradation. Most characterized sRNAs target the 5′ untranslated region (5′ UTR) of the target mRNA to regulate gene expression (33), whereas only a few sRNAs have been reported to bind the CDS, and the majority of these repress gene expression (24,34–36). Thus, these data reveal a comparatively less well-characterized mechanism of sRNA-based regulation. Moreover, to gain a global understanding of the functional implications of MavR-dependent gene expression, we performed RNAseq using *in vitro* conditions that recapitulate EHEC gene expression *in vivo* (15). These data revealed an extensive role for MavR in influencing expression of genes important for growth and virulence during infection. Specifically, deletion of *mavR* affected expression of genes encoding nutrient acquisition, motility (flagella), oxidative stress responses, and AE lesion formation. In agreement with these findings, MavR was required for robust colonization of the mammalian gastrointestinal tract.

## MATERIALS AND METHODS

### Bacterial growth conditions and strain construction

Strains, plasmids and oligonucleotides used in this study are listed in Supplementary Tables S1, S2 and S3, respectively. Bacteria were grown overnight in Lura-Bertani (LB) broth with antibiotics when appropriate (ampicillin [100 μg/ml], streptomycin [100 μg/ml], chloramphenicol [20 μg/ml] and kanamycin [50 μg/ml]). The *mavR*::*cat*, Δ*mavR*, Δ*mavR*Δ*eutR*, Δ*phoB*, Δ*mavR*Δ*phoB, rne*^ΔCTD^ and Δ*mavR rne*^ΔCTD^ strains were generated using Lamda-red recombination (37). To generate non-polar deletion strains the chloramphenicol resistance cassette was resolved with resolvase plasmid pCP20 (all deletion strains except *mavR*::*cat*). As indicated in the text, bacteria were grown in DMEM (Gibco) under aerobic (shaking, atmospheric oxygen) or microaerobic (static, in a 5% CO_2_ incubator) growth conditions.

Arabinose and IPTG inducible expression vectors were generated by using KpnI and HindIII to insert PCR products (*eutR, phoB, flhD, flhC* or *mavR*) into the pBAD/mycHis A or pUCP24 vector. pGEN-mavR was generated using HindIII and NheI to insert the PCR product into pGEN-MCS. p*mavR*-*lux* was generated using PmeI and SnaBI to insert the PCR product into pGEN-luxCDABE. Point mutants, pMS2 and pMS2-*mavR* were generated using the Q5 mutagenesis Kit (NEB). All deletions and plasmids were confirmed by Sanger sequencing. Strains transformed with pBAD/mycHis A vector were grown in the presence of 0.2% arabinose and strains transformed with pUCP24 vector were grown in the presence of 10 μM IPTG.

### Growth curves

Overnight cultures were washed once with PBS, then diluted 1:100 into fresh low-glucose DMEM (Gibco) or M9 minimal medium (50 mM Na_2_HPO_4_·7H_2_O, 20 mM KH_2_PO_4_, 10 mM NaCl, 2 mM MgSO_4_, 100 μM CaCl_2_, 0.4% glycerol, 1 mg/ml thiamine and either 10 mM EA and 150 nM adenosylcobalamin [AdoCbl] or 10 mM NH_4_) and grown at 37°C aerobically. For *in vitro* co-culture experiments, overnight cultures were washed and then diluted 1:200 into the indicated medium. Cultures were plated on LB/streptomycin and LB/chloramphenicol when WT was determined to be at mid-exponential phase in single culture.

### 5′ RACE

5′ RACE was performed as described (38). Briefly, a *mavR*-specific reverse primer (*mavR*3′_qRT_R) was used to reverse transcribe to 5′ end of *mavR* from DNAse-treated RNA. The 5′ end of the transcript was polyadenylated, and the transcript was amplified with a standard primer containing an adapter sequence and a poly-T tract (Q_T_ and Q_o_) and a *mavR* -specific reverse primer (*mavR*5′_qRT_R). This product was further amplified using nested primers and sequenced by Sanger sequencing.

### Northern blotting

Probes were generated using the T7 *in vitro* transcription kit (NEB) incorporating Bio-11-UTP (Fisher) and purified with NucAway spin columns (Invitrogen). Cultures were grown aerobically in M9 with ammonium and 0.2% arabinose to O.D._600_ of 0.3. For bicyclomycin (BCM) assays, 50 μg/ml BCM or vehicle (ethanol) was added for 20 min. For stability assays, an aliquot was removed at time 0, rifampicin was added to a final concentration of 50 μg/ml and additional aliquots were removed at the indicated times. RNA was extracted using the PureLink RNA Mini Kit (Invitrogen). Total RNA concentration was normalized, and samples were mixed with 2× RNA loading buffer (95% formamide, 0.025% SDS, 0.025% bromophenol blue, 0.025% xylene cyanol, 0.5 mM EDTA). After heating to 65°C for 10 min, samples were electrophoresed through a 1.5% MOPS/agarose gel containing 1% formaldehyde. Bands were transferred to Zeta-probe membranes (BioRad) overnight by capillary transfer in 20× SSC. After UV crosslinking the RNA to the membrane, methylene blue was used to visualize the 23S and 16S rRNA bands. Membranes were probed overnight in NorthernMax Prehybridization/Hybridization Buffer (Invitrogen) at 68°C. The membranes were washed twice for 5 min with low stringency wash buffer (0.1% SDS, 2× SSC) and once for 15 min with medium stringency wash buffer (0.1% SDS, 1× SSC) and subjected to the Chemiluminescent Nucleic Acid Detection Module Kit (Thermo Scientific). Northern blots were visualized using a Gel Doc XR + Gel Documentation System (Bio-Rad).

### Luminescence reporter assay

After strains were grown under the indicated condition, a 100 μl aliquot was transferred to a 96-well plate with opaque walls and a transparent bottom. Luminescence readings were accumulated for 10 s by a Wallac Victor 2 plate reader (Perkin Elmer) and normalized to the O.D._600_.

### RT-qPCR

RNA was extracted using the PureLink RNA Mini Kit (Invitrogen) and treated with DNase I (Sigma). RT-qPCR was performed as previously described (24). Briefly, 10 μl reactions containing Power SYBR green master mix (1×, Applied Biosystems), MultiScribe reverse transcriptase (2.5 units, Invitrogen), RNase inhibitor (2 units, Invitrogen), primer mix (0.05 μM each primer) and RNA (50 ng). Reactions were run using the one-step RT-qPCR program on the ABI 7500-FAST sequence detection system and software (Applied Biosystems). Primer sensitivity and specificity were verified by standard curve and melt curve analyses. cDNA generation and amplification were performed as follows: 1 cycle at 48°C for 30 min, 1 cycle at 95°C for 10 min, and 40 cycles at 95°C for 15 s and 60°C for 1 min. Two technical replicates were averaged for analysis by the relative quantification method in which *C*_T_ values were normalized to the reference gene *rpoA* before calculating the ΔΔ*C*_T_ value.

### RNAseq

WT and Δ*mavR* were grown in low glucose DMEM microaerobically for 6 h or aerobically to O.D._600_ 0.5. RNA was extracted using the PureLink RNA Mini Kit (Invitrogen) and DNase treated (Sigma). For the microaerobic cultures, triplicate RNA samples were pooled and sequenced by Novogene. For the aerobic cultures, triplicate (unpooled) RNA samples were barcoded and sequenced by the University of Maryland Genomic Research Core. After rRNA depletion, sequencing libraries were generated and sequenced on an Illumina Novaseq platform. Reads were trimmed and mapped to the EHEC Sakai (NCBI accession NC_002695.2) or EDL933 (NCBI accession NZ_CP008957.1) genome, respectively. HTSeq and the DEGSeq R package were used to determine the FPKM (Fragments Per Kilobase of transcript sequence per Millions base pairs sequenced) and differential gene expression. Differential expression (FC > 2, *P* < 0.05) of selected genes from microaerobic growth conditions was confirmed by RT-qPCR.

### MAPS

To purify the MS2 coat protein, a 1 L culture of BL21 (DE3) cells transformed with pHMM (39) was grown to O.D._600_ 0.7 and induced with 0.5 mM IPTG for 3 h. Cells were pelleted at 5000 RPM for 10 min at 4°C and resuspended in 25 ml of sodium phosphate buffer (50 mM sodium phosphate [pH 7.4], 300 mM NaCl, 10 mM imidazole) containing 250 μl of protease inhibitor cocktail (Sigma) and 100 μg of DNase I (Sigma). The cells were lysed by Emulsiflex C3 (Avestin) and the lysate was clarified by centrifugation at 15 000 RPM for 30 min. The lysate was incubated with Ni-NTA agarose (Qiagen) for 2 h with gentle rocking. The lysate/Ni-NTA agarose mixture was applied to a polypropylene column (Qiagen) and the flow through discarded. After washing the column thrice with 4 ml wash buffer (50 mM sodium phosphate pH 7.4, 300 mM NaCl, 25 mM imidazole), the protein was eluted in 5 ml elution buffer (50 mM sodium phosphate pH 7.4, 300 mM NaCl, 250 mM imidazole) and concentrated using Amicon centrifugal filter units (Millipore). The purified concentrated protein was diluted in 5 ml buffer A (20 mM Tris-HCl pH 8.0, 150 mM KCl, 1 mM MgCl_2_, 10% glycerol) and concentrated again.

MAPS was performed as previously described with a few modifications (31,32). Briefly, cultures were washed and resuspended in buffer A. The cells were lysed by Emulsiflex C3 (Avestin) and the lysate was clarified by centrifugation. MS2-MavR and MS2 RNAs and interacting partners were immunoprecipitated using MS2 coat protein fused to MBP. Eluted RNAs were purified by phenol-chloroform extraction, DNase treated and ethanol precipitated. Sequencing and analysis were completed by the Maryland University Genomic Research Center. Briefly, libraries were prepared using the NEBNext Ultra Directional RNA Library Prep Kit (NEB). Libraries were sequenced using Illumina HiSeq4000 75PE. Reads were mapped to the EHEC EDL933 (NCBI accession NZ_CP008957.1) genome using Bowtie v0.12.7. HTseq and DEseq were used to determine read counts for each gene and calculated fold-enrichment. Target genes were considered to be enriched if > 100 reads mapped to the gene in the MS2-MavR sample and the fold-enrichment was at least 5-fold for the aerobic dataset and at least 2-fold for the microaerobic dataset with a *P* value < 0.1.

### Motility assay

A 1 μl aliquot of WT and Δ*mavR* grown to mid-exponential phase in DMEM was stab inoculated into motility plates (LB with 0.3% agar). Plates were incubated for 6 h at 37°C and halo diameter was measured.

### H_2_O_2_ survival assay

Cultures were grown aerobically in DMEM to mid-exponential phase. Samples were collected immediately prior to and then at 0.5, 1 and 1.5 h after the addition of 5 mM H_2_O_2_. All samples were diluted and plated on LB containing ampicillin immediately after collection. Plates were incubated overnight at 37°C and CFUs were enumerated.

### Mouse colonization

All experiments were approved by the Institutional Animal Care and Use Committee at the University of Virginia School of Medicine. Female 5- to 6-week-old CD-1 mice (Envigo) were infected by oral gavage with a total of 4 × 10^8^ CFUs of bacteria (2 × 10^8^ CFUs of each strain) resuspended in sterile PBS. Fecal samples were collected daily and mice were euthanized 8 days post infection to harvest the ceca and colons. Fecal and tissue samples were homogenized in PBS and CFUs were enumerated on MacConkey agar supplemented with streptomycin or chloramphenicol. The competitive index was calculated as the ratio of *mavR*::*cat* to WT normalized to the inoculum.

### Fluorescent actin staining (FAS) assay

HeLa cells were seeded at a cell density of 5 × 10^5^ cells/well on coverslips in a 12-well dish. The following day, HeLa cells were washed and infected with the indicated EHEC strains at a multiplicity of infection (M.O.I.) of 100 in low-glucose DMEM. Infected HeLa cells were incubated at 37°C with 5% CO_2_ for 6 h, replacing the media at 3 h. Coverslips were washed thrice with PBS and cells were fixed with 0.75% formaldehyde for 20 min. Cells were permeabilized with 0.2% Triton-X for 6 min and stained with 1 μg/ml fluorescein isothiocyanate (FITC)-phalloidin for 20 min at 37°C to visualize actin. After RNase A treatment (1 mg/ml for 10 min), coverslips were stained with 4 μg/ml DAPI to visualize DNA. Pedestals were enumerated for at least 98 HeLa cells per condition.

### Western blotting

Bacterial overnight cultures were diluted 1:100 into M9 minimal medium and grown aerobically to an O.D._600_ of 0.4 (Supplementary Figure S5) or diluted 1:100 into DMEM and grown microaerobically for 6 h. Cultures were pelleted by centrifugation and resuspended in sterile PBS. After the addition of SDS sample buffer, samples were boiled for 10 min and electrophoresed through SDS-PAGE gels and transferred to PVDF membranes (BioRad). Membranes were blocked with 5% dry milk in TBST, washed with TBST thrice and incubated with primary anti-His (1:1000, Cell Signaling), anti-DnaK (1:10 000, AbCam), anti-GAPDH (Invitrogen) or anti-EspA (1:7500, Vanessa Sperandio) antibodies for 1 h at room temperature. After three TBST washes, membranes were incubated with secondary anti-mouse or anti-rabbit antibodies conjugated to horse radish peroxidase for 30 min at room temperature. After TBST washes, ECL (PerkinElmer) was added to the membranes and bands were visualized on a Gel Doc XR + Gel Documentation System.

### Electrophoretic mobility shift assays (EMSAs)

T7 *in vitro* transcription (NEB) was performed for *eutR* and *mavR* incorporating Bio-11-UTP (Fisher) where indicated. Transcripts were purified using NucAway Spin Columns (Invitrogen). Purified transcripts were incubated at the indicated concentrations in 1× structure buffer (Ambion RNase T1 kit), 2 ng/µl yeast RNA (Ambion) and brought to a total volume of 20 μl with RNase-free water. Samples were incubated at 85°C for 3 min followed by 20 min at 37°C. Following the addition of 5× RNA loading dye (50% glycerol, 0.1% bromophenol blue), samples were subjected to electrophoresis on 5% native TBE gels using 1× TBE as running buffer. Electrophoresed samples were transferred by capillary action to Zeta-probe membranes (BioRad) in 20× SSC. After UV crosslinking the RNA to the membrane, the membranes were subjected to the Chemiluminescent Nucleic Acid Detection Module Kit (Thermo Scientific). EMSAs were visualized using a Gel Doc XR + Gel Documentation System.

### RNase E cleavage assay

The N-terminus of RNase E (AAs 1–527, N-Rne) was cloned into pBAD/mycHis A for protein purification as described above (see MAPS methods) with the modification that expression was induced with 0.2% arabinose. *In vitro* transcribed *eutR* was biotinylated on the 3′ end using the Pierce RNA 3' End Biotinylation Kit (Thermo). Transcripts were incubated at the indicated concentrations in 1× structure buffer (Ambion RNase T1 kit), 2 ng/μl yeast RNA (Ambion), and brought to a total volume of 10 μl with RNase-free water. Samples were incubated at 85°C for 3 min followed by 20 min at 37°C. RNase E was added to a final concentration of 7.5 μM and samples were incubated at 37°C for an additional 30 or 60 min. Then, 2× denaturing loading buffer (95% formamide, 0.025% SDS, 0.025% bromophenol blue, 0.025% xylene cyanol, 0.5 mM EDTA) was added and samples were subjected to electrophoresis on 10% TBE-urea gels at 150 V. After electrophoretic transfer (50 V, 2 h) to Zetaprobe membrane, the membranes were crosslinked. Membranes were developed and visualized as previously described.

### Bioinformatic analyses

All programs used for bioinformatic analyses are listed and referenced in Supplementary Table S4.

## RESULTS

### MavR is a pathogen-specific sRNA

**Figure 1.**
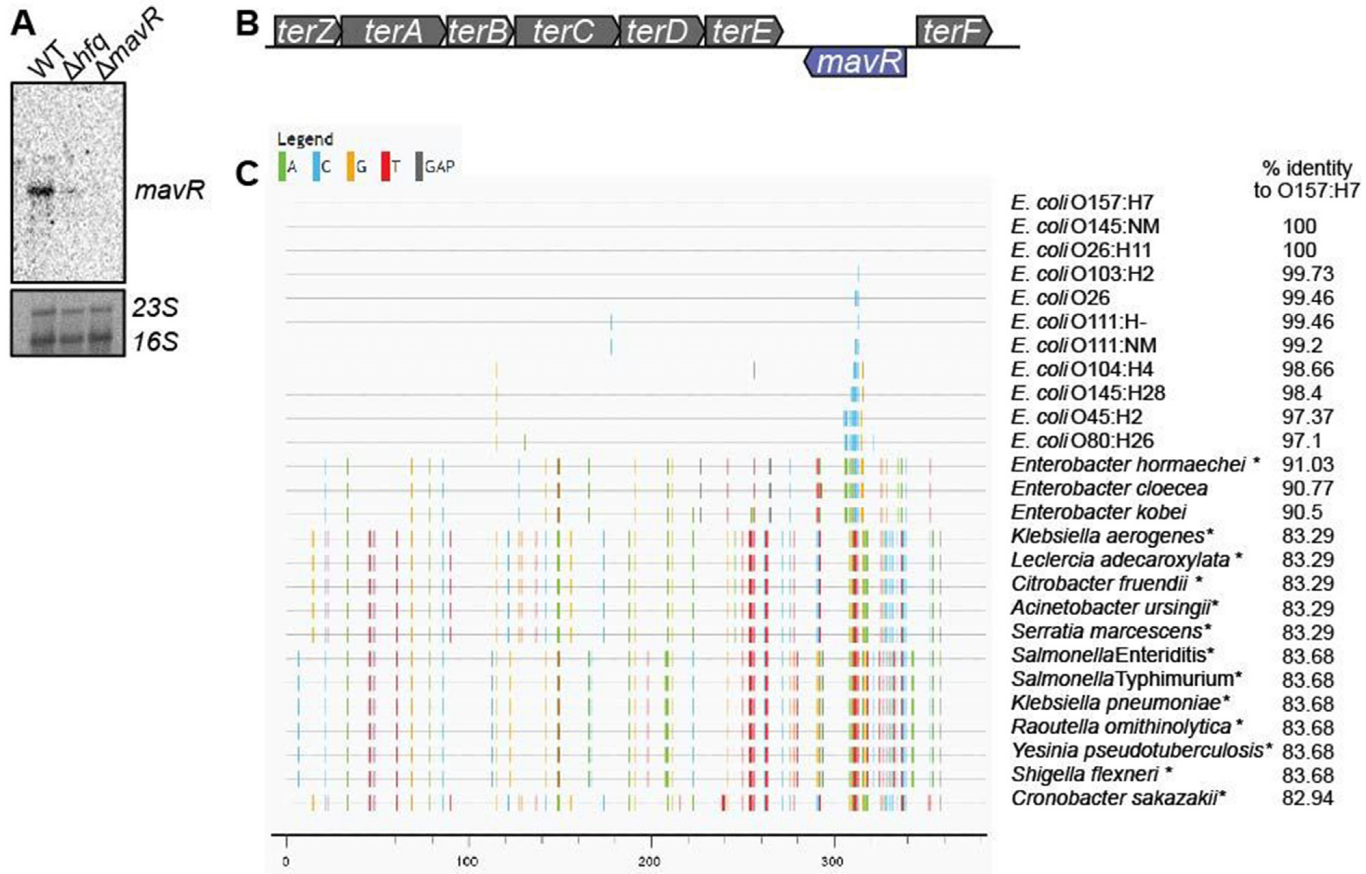
MavR is a pathogen-specific sRNA. (**A**) Northern blot of MavR in WT, Δ*hfq*, and Δ*mavR* grown under microaerobic conditions. 23S and 16S rRNA are shown as loading controls; *N* = 2. (**B**) Schematic of the genomic context of *mavR*. (**C**) Genomic alignment of MavR from the indicated bacteria. Each vertical bar represents a base mismatch with respect to the *E. coli* O157:H7 sequence. Therefore, areas without vertical bars represent more highly conserved sequences to *E. coli* O157:H7 compared to regions with vertical bars. Asterisks indicate genomes in which *mavR* is plasmid- encoded. Percent identity is also indicated on the right side.

MavR is an Hfq-dependent sRNA that is produced under conditions that promote EHEC virulence gene expression (19,28) (Figure 1A). MavR is encoded within O-island 48 in the reverse orientation of genes encoding tellurite resistance (*ter*) (Figure 1B). O-islands are DNA regions present in EHEC but absent in *E. coli* K-12 (27). Further analyses revealed that MavR is conserved in diarrheagenic *E. coli* as well as in other pathogenic *Enterobacteriaceae* (Figure 1C) but absent from nonpathogenic bacteria. Notably, the genomic organization of *mavR* within the *ter* locus is maintained in all bacteria that encode this sRNA.

RNAseq analyses indicated that MavR is an ∼370 bp sRNA ((28) and data herein). To examine *mavR* transcriptional control, we performed 5′ RACE and *in silico* analyses using Bprom, which suggested that *mavR* contains a housekeeping sigma 70 recognition sequence in the promoter (Supplementary Figure S1A). In support of constitutive MavR expression, transcriptional analyses indicated that *mavR* is similarly expressed throughout growth as well as under microaerobic and aerobic conditions (Supplementary Figure S1B–D). To study MavR-dependent gene expression, we generated a *mavR* deletion in EHEC strain 86–24 (Figure 1A) and performed genome sequencing (Illumina and PacBio) to confirm that strain 86–24 encodes *mavR* only in single copy (EHEC strain EDL933 encodes two copies) as well as to ensure no off-target effects (e.g., spontaneous mutations) occurred. We also confirmed that the *mavR* deletion did not affect expression of surrounding genes (Supplementary Figure S2).

### Global mapping of MavR targets

To identify MavR targets, we performed MS2 affinity purification coupled to RNA sequencing (MAPS) (31,32). For these experiments, we fused the MS2 aptamer to the 5′ end of *mavR*, and this chimera was cloned under control of an arabinose-inducible (pBAD) promoter, generating pBAD-MS2::MavR. The MS2::MavR construct is functional as it complemented gene expression in the Δ*mavR* strain (Figure 7D). Affinity purification was performed using Δ*mavR* carrying the pBAD-MS2::MavR or the empty pBAD-MS2 construct to eliminate competition with native copies of MavR and to control for false positives interacting with the MS2 aptamer. Oxygen is an important signal that influences EHEC gene expression (24,40); therefore, to more comprehensively map the MavR interactome, MAPS was performed under aerobic and microaerobic growth conditions.

**Figure 2.**
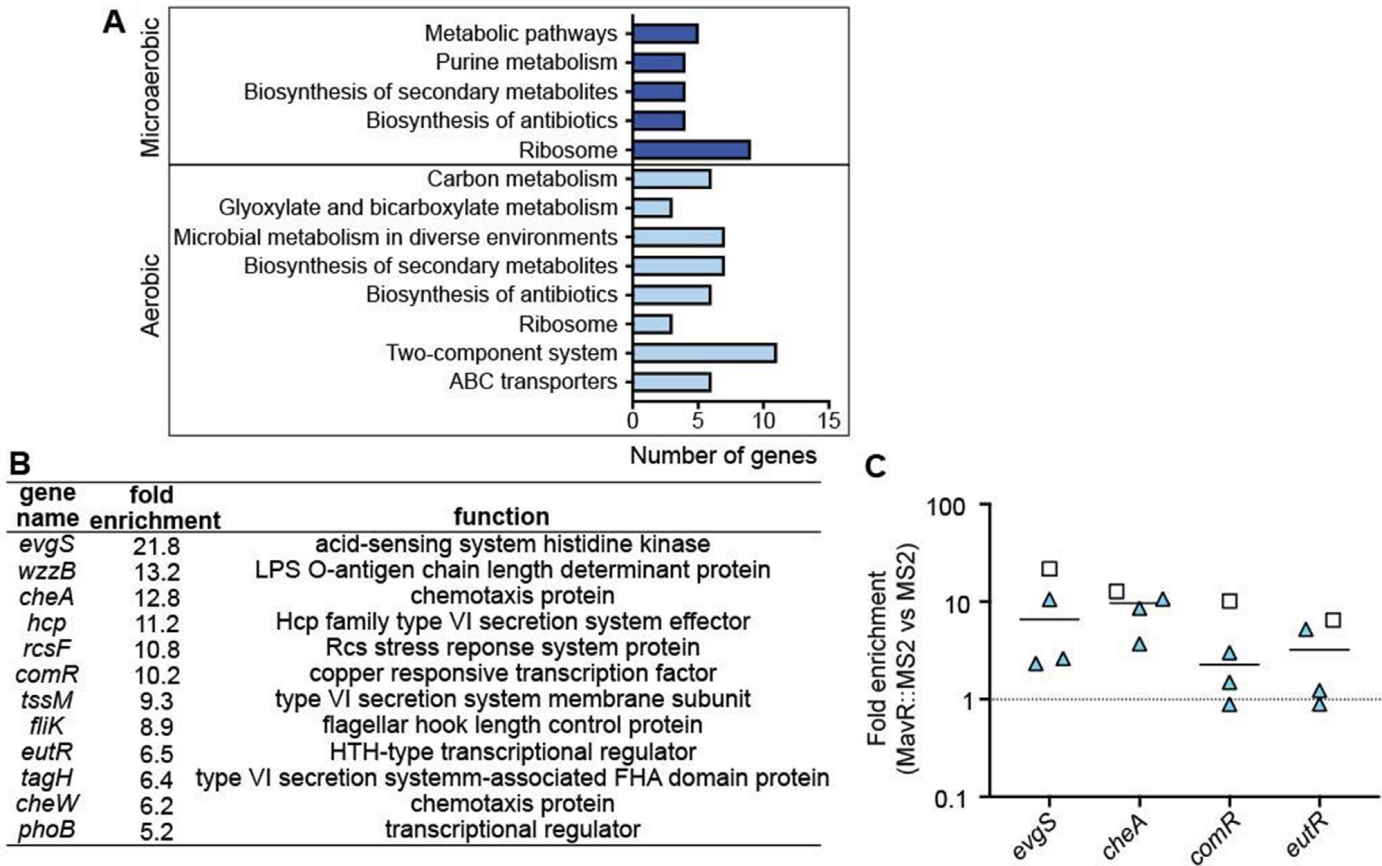
Overview of MAPS data. (**A**) Pathway analysis of the MAPS data. (**B**) Table of transcripts encoding virulence-associated factors enriched in the MAPS data set. (**C**) RT-qPCR (triangles) of transcripts identified as enriched by MAPS (squares) from MS2::MavR affinity purified RNAs normalized to MS2 affinity purified RNAs.

We identified 36 transcripts enriched in the microaerobic dataset and 185 transcripts enriched in the aerobic dataset (>100 mapped reads, *P* < 0.1). Kegg pathway enrichment analyses revealed enrichment of diverse processes, including metabolism, translation, motility/chemotaxis and gene regulation (Figure 2A). Enriched targets also included several genes previously reported to contribute to EHEC virulence (Figure 2B). We repeated the MS2 affinity purification assay and validated targets via RT-qPCR. In agreement with the MAPS data, we measured enrichment of the *evgS*, *cheA*, and *comR* transcripts in the MS2::MavR affinity purification compared to the MS2 affinity purification (Figure 2C). Enrichment of the *eutR* transcript was more variable (Figure 2C), most likely because this assay was performed under conditions in which *eutR* is expressed at low levels (see subsequent results sections and the discussion section).

### MavR interacts with the 3′ region of the eutR coding sequence to promote expression

**Figure 3.**
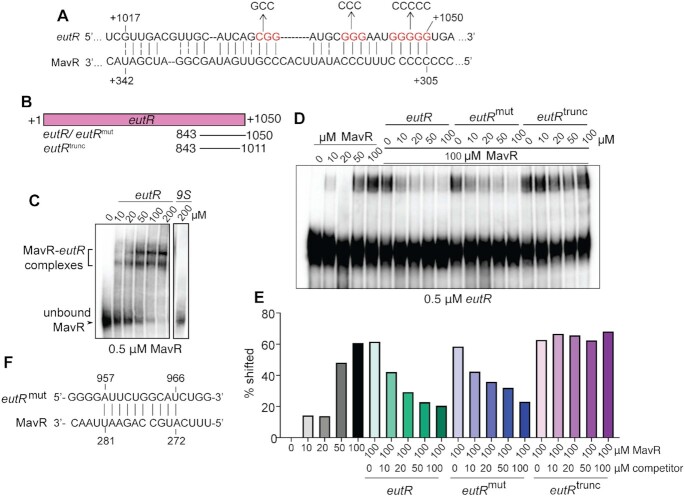
MavR binds the *eutR* transcript. (**A**) Predicted MavR-*eutR* RNA base-pairing. Point mutations to generate the disrupted alleles in the *eutR*^mut^ transcript are indicated. Numbering on the *eutR* sequence indicate nucleotides after the translation start site. Numbering on the MavR sequence indicate nucleotides after the transcription start site. (**B**) Schematic showing the *in vitro* transcribed RNAs used for the EMSAs. (**C**) RNA EMSA of labeled MavR and *eutR* transcripts. 9S precursor RNA is the negative control. (**D**) Competition RNA EMSA of labeled *eutR* and unlabeled MavR transcripts competed with indicated unlabeled *eutR* transcripts. (**E**) Quantification of competition RNA EMSA in (D). (**F**) Predicted interaction between *eutR^mut^* and MavR transcripts.

To investigate MavR-dependent gene regulation in more detail, we focused on *eutR*, which was identified as a putative target by MAPS. EutR was initially characterized nearly 30 years ago as the transcriptional activator of the *eut* (EA utilization) locus that is required for EA metabolism (41,42). EA metabolism enhances pathogen growth during host infection (43–48). Recent work in our lab established that EutR also regulates virulence genes, including T3SS expression in EHEC, *Salmonella enterica* serovar Typhimurium and *Citrobacter rodentium* (16,43,47–50). To examine MavR interaction with the *eutR* transcript, we performed bioinformatic analysis using IntaRNA (51–54) to identify potential interaction sites. These analyses predicted an interaction between MavR and the 3′ region of the *eutR* coding sequence (CDS) (Figure 3A, free energy value of -12.16). To test this prediction, we performed RNA electrophoretic mobility shift assays (EMSAs) using *in vitro* transcribed and biotinylated MavR and increasing amounts of the 3′ region of the *eutR* transcript (Figure 3B) or 9S (negative control) transcript. Upon addition of the *eutR* transcript, we measured a shift in the MavR RNA, indicating direct base pairing, whereas no shift occurred upon addition of the 9S transcript (Figure 3C).

We repeated the EMSAs using biotinylated *eutR* and increasing concentrations of the MavR transcript. These data were consistent with the previous EMSA results, as we measured a shift in the labeled *eutR* transcript upon addition of MavR to the reactions (Figure 3D–E). Next, we performed competition EMSAs using labeled *eutR* and increasing amounts of unlabeled wildtype *eutR*, mutated *eutR* or truncated *eutR* (as indicated in Figure 3B). The *eutR* transcript effectively competed with labeled *eutR* for MavR binding. To substantiate these data, we generated mutations in the *eutR* transcript (shown in red in Figure 3A, which were designed to disrupt G-C pairing) and repeated the EMSAs. Surprisingly, the mutated *eutR* transcript (*eutR*^mut^) was able to interact with MavR, as indicated by the decreased intensity of shifted *eutR* transcript (Figure 3D–E). Subsequent *in silico* analysis revealed sequence complementarity between MavR and *eutR*^mut^ transcripts (Figure 3F), suggesting we simply changed the binding site. This conclusion is supported by previous work which showed that altering of nucleotides in an sRNA-RNA binding site can shift target binding and include interactions with sequences that exhibit complementarity to the mutated binding site (55). Therefore, we generated a truncated *eutR* transcript (*eutR*^trunc^) to remove the entire 3′ end of the *eutR* transcript. The truncated transcript did not compete for MavR binding as there was virtually no difference in the amount of shifted *eutR* in the absence or presence of *eutR*^trunc^ (Figure 3D–E). Collectively, these data support MavR interaction with the 3′ region of the *eutR* transcript.

**Figure 4.**
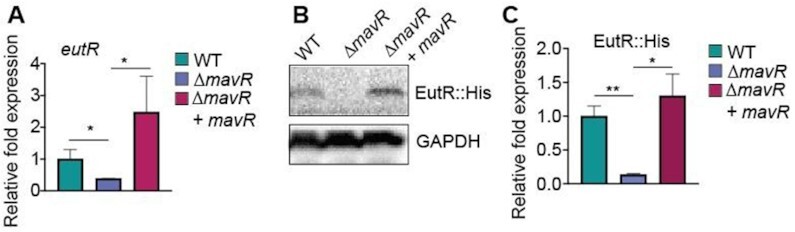
MavR promotes expression of *eutR*/EutR. (**A**) RT-qPCR of *eutR* transcript levels expressed from pBAD-*eutR*::His in WT, Δ*mavR*, and Δ*mavR*+ pUCP24-*mavR*. WT and Δ*mavR* carry the empty vector, *N* = 3. (**B**) Western blot of EutR::His in WT, Δ*mavR* and Δ*mavR*+ pUCP24-*mavR*. GAPDH is the loading control. (**C**) Quantification of EutR::His expression in WT, Δ*mavR* and Δ*mavR*+ pUCP24-*mavR*, *N* = 9. Bars represent the mean and error bars indicate standard error of the mean (SEM). ns *P* > 0.05, * *P* < 0.05, ** *P* < 0.01 (Student’s two-sample *t*-test).

Next, we examined the effect of MavR on EutR expression. For these experiments, *eutR* was fused to a Myc-His tag and cloned under the control of an arabinose-inducible vector to specifically assay post-transcriptional regulation. The resulting plasmid (pEutR::His) was introduced into WT and Δ*mavR*. EutR::His expression was decreased in Δ*mavR* compared to WT (Supplementary Figure S3A,B). To substantiate MavR regulation of EutR expression, we utilized a two plasmid-based assay in which WT and Δ*mavR* were transformed with pEutR::His or pEutR::His and pMavR. Dual plasmid systems are routinely used to monitor the effects of a sRNA on the target mRNA and enables the specific effects of the sRNA on the target RNA to be assessed (19,24,30,56,57). RT-qPCR and western analysis confirmed MavR positively regulated *eutR/*EutR expression, as expression was decreased in Δ*mavR*, and this defect was rescued when MavR was expressed *in trans* (Figure 4A–C). Collectively, these data indicated that MavR positively regulates EutR expression.

### MavR is required for robust expression of the *eut* locus and maximal EA-dependent growth

**Figure 5.**
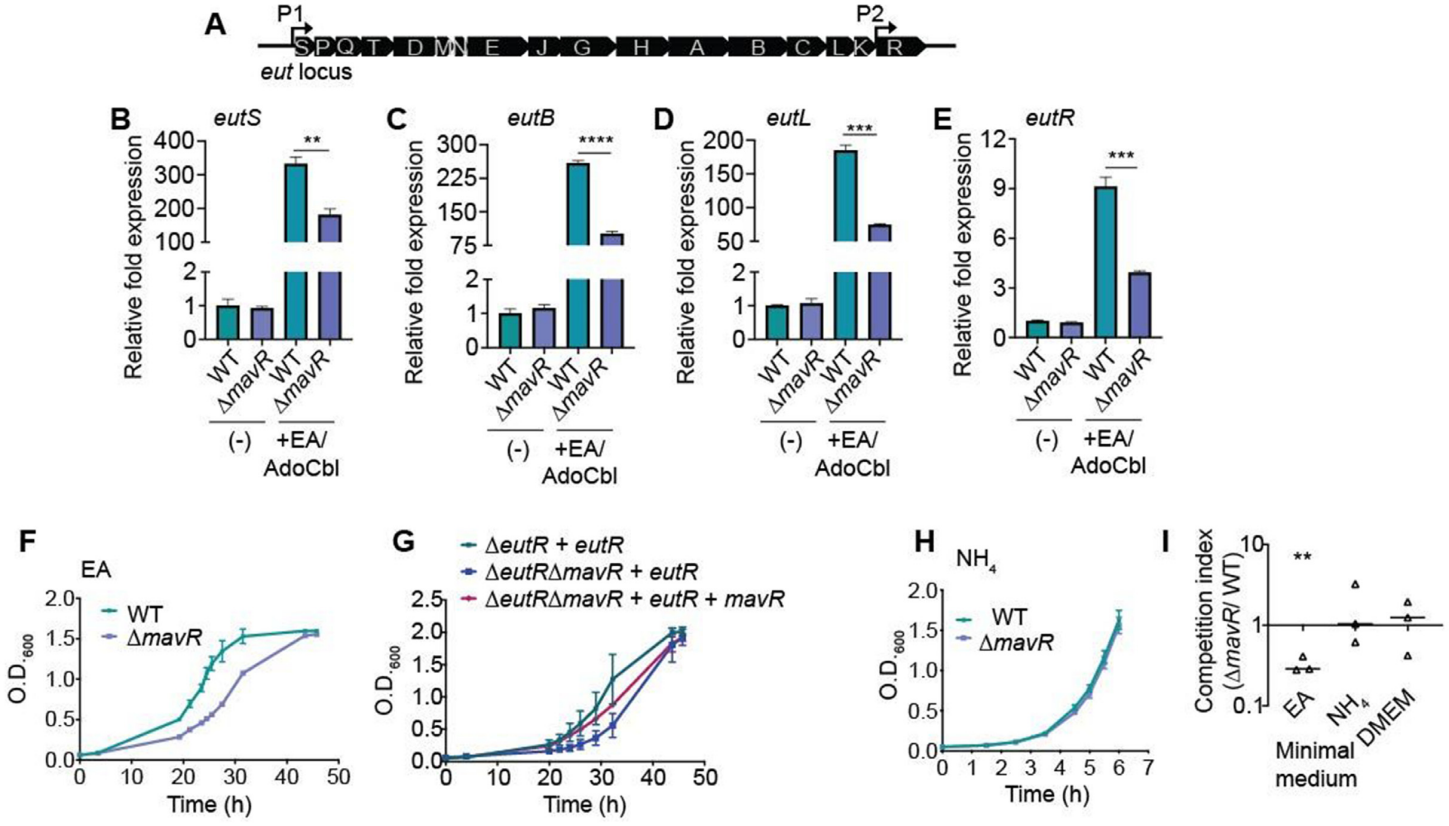
MavR is required for robust expression of the ethanolamine utilization (*eut*) locus and EA utilization. (**A**) Schematic of the *eut* locus. (**B**) RT-qPCR of *eutS* expression in WT and Δ*mavR* grown without or with ethanolamine (EA) and adenosylcobalamin (AdoCbl) supplementation. (**C**) RT-qPCR of *eutB* expression in WT and Δ*mavR* grown without or with EA and AdoCbl supplementation. (**D**) RT-qPCR of *eutL* expression in WT and Δ*mavR* grown without or with EA and AdoCbl supplementation. (**E**) RT-qPCR of *eutR* expression in WT and Δ*mavR* grown without or with EA and AdoCbl supplementation. (**F**) Growth curves of WT and Δ*mavR* in minimal medium containing EA as the sole nitrogen source. (**G**) Growth curves of Δ*eutR* + pBAD-*eutR*, Δ*eutR*Δ*mavR* + pBAD-*eutR* and Δ*eutR*Δ*mavR*+ pBAD-eutR and pUCP24-*mavR* in minimal medium containing EA as the sole nitrogen source. (**H**) Growth curves of WT and Δ*mavR* in minimal medium containing NH_4_ as the sole nitrogen source. (**I**) Competition assay between WT and Δ*mavR::cat* grown in minimal medium containing EA or NH_4_ as the sole nitrogen source and in DMEM. Bars represent the mean and error bars indicate SEM. *N* = 4, ** *P* < 0.01, *** *P* < 0.001, **** *P* < 0.0001 (Student’s two-sample *t*-test).

The *eut* locus is comprised of 17 genes (Figure 5A) that encode proteins that function in the transport and catabolism of EA as well as a protein microcompartment that contains toxic breakdown products of EA metabolism (41,42,58–61). EutR is encoded by the last gene in the *eut* locus and is required for transcriptional activation of the entire locus (41,42). *eutR* is constitutively expressed at low levels from an internal P2 promoter. EutR binds to the primary P1 promoter upstream of *eutS*, and in the presence of EA and adenosylcobalamin (AdoCbl), EutR activates transcription. This results in robust expression of the entire operon, including readthrough of the P2 promoter and positive autoregulation (42,62). Because MavR promotes EutR expression, we reasoned that Δ*mavR* would be impaired for *eut* expression. To test this idea, we examined native expression of *eutS, eutB, eutL* and *eutR* (as representative genes in the beginning, middle and end of the locus) (Figure 5A) after growth without or with EA and AdoCbl. No differences in *eut* gene expression were measured when EHEC was grown without EA and AdoCbl supplementation; however, under *eut*-inducing conditions, we measured a 2- to 3-fold decrease in expression of all genes in Δ*mavR* compared to WT (Figure 5B–E). Consistent with MavR being a Hfq-dependent sRNA, there was a similar 3-fold decreases in *eut* gene expression in Δ*hfq* compared to WT when these strains were grown in DMEM supplemented with EA and AdoCbl (Supplementary Figure S4A–D). To determine whether EA or AdoCbl influenced post-transcriptional *eut* expression (as has been reported for Firmicutes (63,64)), we measured EutR::His expression after growth in medium without supplementation or with EA, AdoCbl, or EA and AdoCbl. In all conditions, EutR::His was detected at lower levels in Δ*mavR* compared to WT (Supplementary Figure S5). Moreover, no differences in EutR::His expression were detected in the WT strain regardless of EA and/or AdoCbl, and similarly, these molecules did not affect EutR::His levels in Δ*mavR* (Supplementary Figure S5), indicating that MavR regulates EutR expression independently of EA and AdoCbl.

To functionally test the consequence of MavR-dependent *eut* expression, we measured growth of WT and Δ*mavR* in minimal medium containing EA as the sole nitrogen source. We measured a longer lag phase of Δ*mavR* compared to WT (Figure 5F). To confirm the role of MavR on EA utilization, we utilized the dual plasmid-based assay (described above) in which Δ*eutR* or Δ*eutR*Δ*mavR* was transformed with pEutR::His or pEutR::His and pMavR. MavR expression rescued growth of the Δ*eutR*Δ*mavR* strain to near WT (Δ*eutR* + pEutR::His) levels (Figure 5G). Importantly, this growth defect was specific to EA utilization, as WT and Δ*mavR* grew similarly in minimal medium containing ammonium as the sole nitrogen source (Figure 5H) as well as in DMEM (Supplementary Figure S6A,B). In agreement with single strain growth assays, during growth in co-culture, Δ*mavR::cat* (unresolved deletion strain) was significantly outcompeted by WT in minimal medium supplemented with EA, but was recovered at similar levels to WT during growth in minimal medium supplemented with ammonium or in DMEM (Figure 5I). Collectively, these data demonstrate that MavR is necessary for maximal *eut* expression and EA utilization.

### MavR stabilizes the *eutR* transcript

sRNAs regulate gene expression typically by enhancing or preventing translation initiation (21). However, sRNAs may also antagonize Rho-dependent transcription termination or affect stability of the target transcript (65,66). Because of the proximity of the MavR-*eutR* binding site to the 3′ end of the transcript as well as that MavR influenced *eutR* transcript levels, we excluded translation initiation as a potential mechanism. To test whether MavR affected Rho-dependent transcription termination, we added the Rho inhibitor bicyclomycin (BCM) (67) to cultures of WT and Δ*mavR* and assessed *eutR* levels by northern analysis. The *eutR* transcript levels were decreased in Δ*mavR* compared to WT regardless of the addition of BCM to the culture media (Supplementary Figure S7A). We repeated these experiments to measure endogenous *eut* expression following growth of WT and Δ*mavR* under *eut*-inducing conditions. Native *eut* expression was ∼2-fold reduced in Δ*mavR* compared to WT without or with BCM treatment (Supplementary Figure S7B). These data indicated that MavR does not antagonize Rho to promote *eutR*/ *eut* expression.

**Figure 6.**
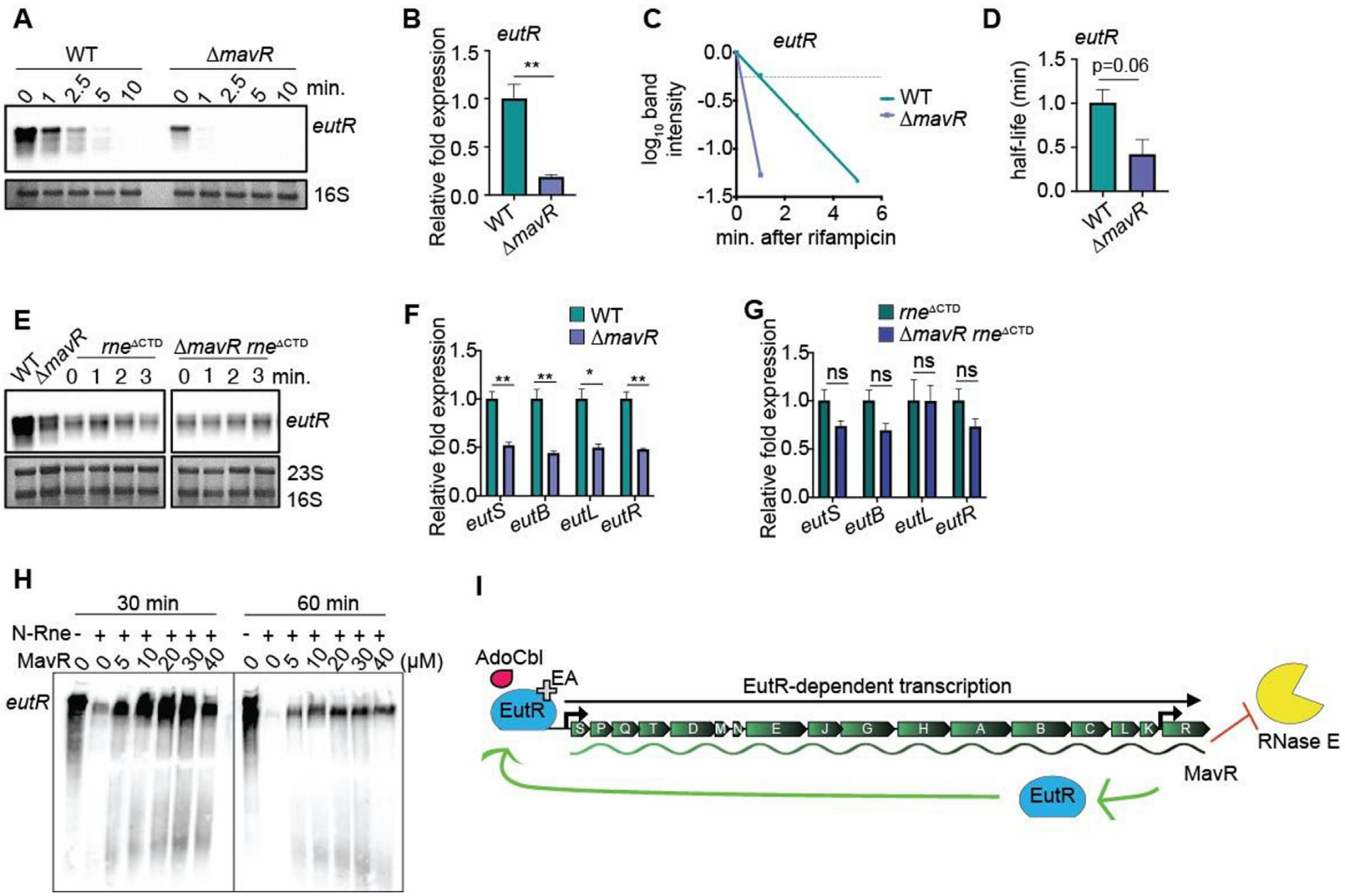
MavR stabilizes the *eutR* transcript. (**A**) Northern blot of *eutR* transcripts expressed from pBAD-*eutR*::His in WT and Δ*mavR* at indicated time points before or after addition of rifampicin. 16S rRNA is the loading control. (**B**) Relative quantification of *eutR* expression in WT and Δ*mavR* prior to addition of rifampicin (time 0, in panel (A)), *N* = 3. (**C**) Decay curves of *eutR* RNA in WT and Δ*mavR*. The signal obtained at 0 min. was set to 1 for each strain, and the amount of RNA remaining at each timepoint was plotted on the *y*-axis versus time on the *x*-axis. The time point at which 50% of the *eutR* mRNA had been decayed (dashed line) was calculated to determine the half-life (*t*_1/2_), *N* = 3. (**D**) Half-life of *eutR* expressed from pBAD-*eutR*::His. *N* = 3. (**E**) Northern blot of *eutR* transcripts expressed from pBAD-*eutR*::His in WT, Δ*mavR, rne*^ΔCTD^ and Δ*mavR rne*^ΔCTD^. (**F**) RT-qPCR of *eut* gene expression in WT and Δ*mavR*, *N* = 3. (**G**) RT-qPCR of *eut* gene expression in *rne*^ΔCTD^ and Δ*mavR rne*^ΔCTD^, *N* = 3. For (B), (D), (F) and (G), bars represent the mean and error bars indicate SEM. ns *P* > 0.05, * *P* < 0.05, ** *P* < 0.01 (Student's two-sample *t*-test). (**H**) *In vitro* cleavage of *eutR* by N-Rne in the absence or presence of MavR. (**I**) Model: MavR interaction with the *eutR* transcript protects *eutR* from RNase E-mediated degradation. Stabilized *eutR* transcripts result in maximal EutR expression and EutR-dependent activation of *eut* expression.

To examine the effect of MavR on *eutR* stability, we grew WT and Δ*mavR* transformed with pEutR::His. After growth, cultures were treated with rifampicin to inhibit transcription initiation, and RNA was extracted from sample aliquots immediately prior to and at indicated timepoints following rifampicin treatment. *eutR* transcript abundance and stability was then determined by northern blot analysis. At time 0, *eutR* transcript levels were decreased nearly 4-fold in Δ*mavR* compared to WT (Figure 6A,B), which was consistent with data shown in Figures 4A and 5E. Furthermore, the *eutR* transcript was ∼3–4 fold less stable in Δ*mavR* (half-life ∼0.25 min) as compared to WT (half-life ∼1 min) (Figure 6A, C and D). Because the His tag could influence the stability and/or processing of the *eutR* transcript, we repeated these experiments using WT and Δ*mavR* transformed with pEutR (expressed from pBAD24). These data revealed only a modest decrease in *eutR* transcript stability in Δ*mavR* compared to WT (Supplementary Figure S8A,B). We were unable to demonstrate that MavR affects the half-life of the native *eutR* transcript (data not shown), possibly because *eutR* is the last gene in the long *eut* operon (68). Because the data shown in Figure 6A–D suggested that MavR influences *eutR* stability, we further interrogated this hypothesis, as described below.

RNase E initiates degradation of most transcripts in the *Enterobacteriaceae* (69–73). A previous, unbiased analysis reported that the *eutR* transcript co-immunoprecipitated with RNase E in EHEC strain Sakai (74). Therefore, we investigated the hypothesis that MavR protects the *eutR* transcript from RNase E-dependent degradation. Because RNase E is encoded by the essential *rne* gene, we first generated a truncated RNase E protein that lacks the C-terminal domain (CTD) (75,76) in WT and Δ*mavR* (generating *rne*^ΔCTD^ and Δ*mavR rne*^ΔCTD^). The CTD functions as a scaffold in assembly of the degradosome. Bacteria that lack this domain are viable but accumulate RNA processing intermediates (76–78). We performed northern analysis to compare *eutR* expression in WT, Δ*mavR, rne*^ΔCTD^ and Δ*mavR rne*^ΔCTD^ strains (as an experimental control, *eutR* expression was also measured in WT and Δ*mavR*, Figure 6E, left side). Although total *eutR* levels were decreased in the *rne* background compared to WT, this truncation ablated differences in *eutR* expression between *rne*^ΔCTD^ and Δ*mavR rne*^ΔCTD^ strains at time 0 (Figure 6E). Moreover, the RNase E truncation stabilized the *eutR* transcript as no measurable degradation of the *eutR* RNA occurred during the course of the experiment in either *rne*^ΔCTD^ or Δ*mavR rne*^ΔCTD^ (Figure 6E). As a complementary approach, we examined whether limiting RNase E activity would rescue endogenous *eut* expression in Δ*mavR* compared to WT. In agreement with the plasmid-based data, there was no significant difference in *eut* gene expression between *rne*^ΔCTD^ and Δ*mavR rne*^ΔCTD^ (Figure 6F and G).

Next, we performed *in vitro* RNase E cleavage assays. The rationale for this experiment was two-fold. First, this experiment would indicate whether the lack of degradosome in the *rne*^ΔCTD^ strains vs RNase E catalytic activity in the previous assays was responsible for rescue of *eutR/eut* expression. Second, the *in vitro* cleavage assay experiment also addresses the role of MavR *per se* in antagonizing RNase E-dependent degradation of the *eutR* transcript. For these experiments, we expressed and purified the N-terminal region of RNase E (N-Rne) that contains the catalytic domain and possesses full cleavage activity (79,80). Upon addition of N-Rne, the *eutR* transcript was partially degraded within 30 min and nearly undetectable after 60 min. However, the addition of MavR to the reactions prevented RNase E hydrolysis (Figure 6H). These data demonstrate that MavR is sufficient to protect the *eutR* transcript from RNase E-mediated degradation independent of additional regulatory factors. Collectively, these data support a model in which MavR protects *eutR* from RNase E mediated cleavage to promote *eut* expression (Figure 6I).

### MavR influences expression of diverse genes important for EHEC fitness and virulence

**Figure 7.**
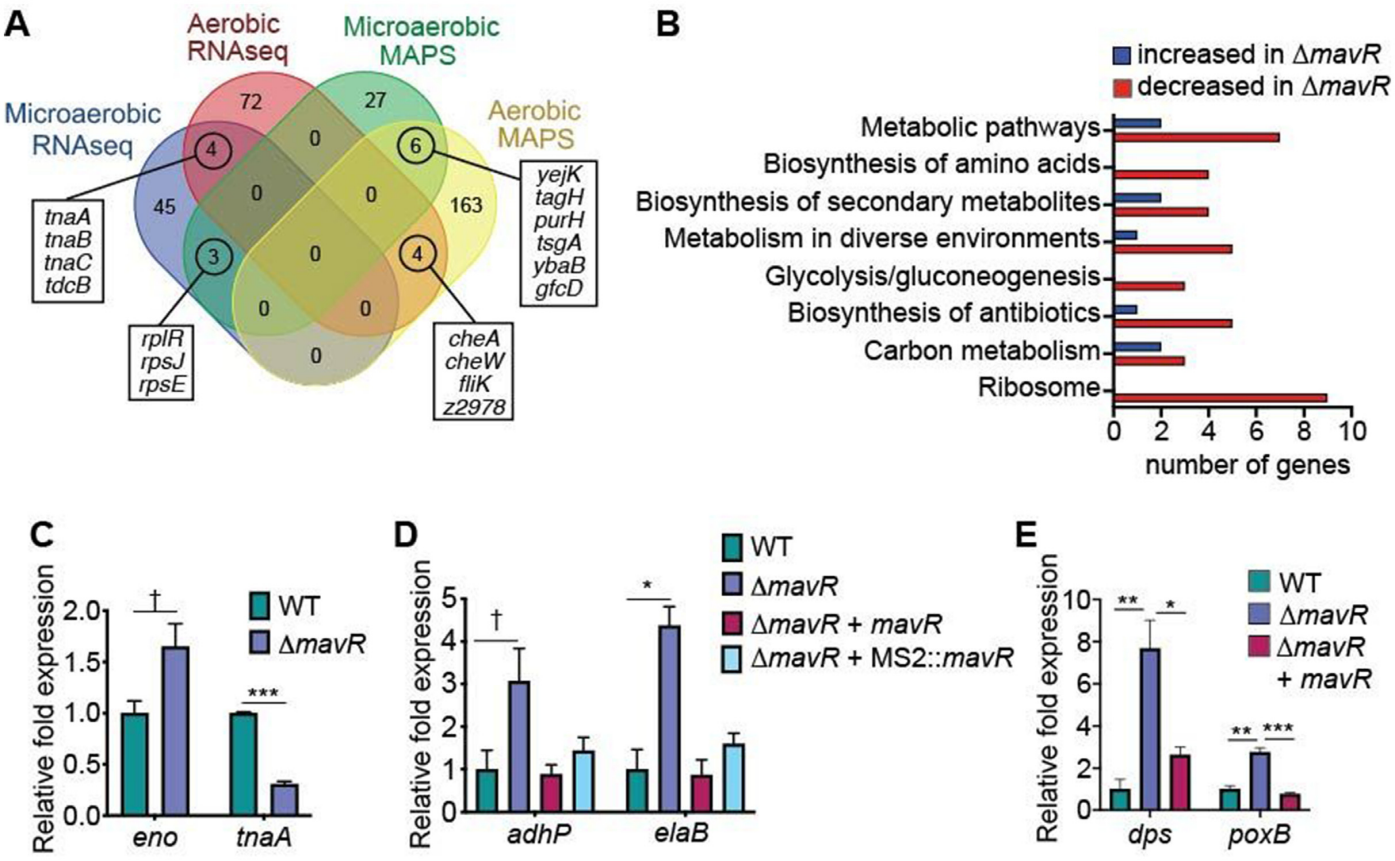
Overview of MavR-dependent gene expression. (**A**) Comparison of MAPS and RNAseq datasets. (**B**) Pathway analysis of microaerobic RNAseq data. (**C**) RT-qPCR of indicated genes identified as differentially expressed in the microaerobic RNAseq data set in WT and Δ*mavR*. (**D**) RT-qPCR of indicated genes in WT, Δ*mavR*, Δ*mavR* + pBAD-*mavR* and pBAD-MS2::*mavR*. WT and Δ*mavR* carry the empty vector, *N* = 3. (**E**) RT-qPCR of indicated genes in WT, Δ*mavR* and Δ*mavR*+ pGEN-*mavR*. WT and Δ*mavR* carry the empty vector. Bars represent the mean and error bars indicate SEM; * *P* < 0.05, ** *P* < 0.01, *** *P* < 0.001, † *P* < 0.1 (Student’s two-sample *t*-test).

To globally assess the biological impact of MavR on EHEC gene expression, we performed RNAseq using RNA purified from WT and Δ*mavR* grown under aerobic and microaerobic conditions. There were no putative targets/ differentially expressed genes common to all of the MAPS and RNAseq data sets (Figure 7A). However, we identified a handful of shared genes between the aerobic MAPS and RNAseq data, the microaerobic MAPS and RNAseq data sets, as well as transcripts that were enriched in both the aerobic and microaerobic MAPS. Under aerobic growth, MavR affected expression of 87 genes (fold change ≥ 2-fold and *P* ≤ 0.05). Of the differentially expressed genes, 11 genes were increased in Δ*mavR* compared to WT. These genes encode metabolic regulators and enzymes (*tcdR, tcdB, wcaK, ccmE, dcuC*), a putative diguanylate synthase *(cdgI*), as well as hypothetical/ uncharacterized proteins (*Z2249, Z0326, Z2717/ydiL, Z2395* and *Z1866*). Genes that were decreased in Δ*mavR* compared to WT primarily encode flagella and motility (detailed in the next section).

Under microaerobic conditions, MavR impacted expression of 52 genes. Although only three overlapping transcripts were identified in the MAPS and RNAseq data sets, Kegg pathway enrichment analyses were consistent between the data in revealing enrichment of metabolic- and ribosome-associated pathways among the differentially regulated genes (Figure 7B). We performed RT-qPCR using RNA harvested from a distinct set of biological replicates to confirm MavR-dependent expression of genes encoding metabolic enzymes involved in glycolysis and amino acid metabolism as well as *elaB* that plays a role in stress responses (Figure 7C,D).

The transcriptomic data also revealed that genes that encode the T6SS and the T3SS, which contribute to or are required for EHEC virulence, respectively (1,81,82) were decreased in Δ*mavR* compared to WT (Supplementary Figure S9 and detailed in a subsequent section) and that genes encoding oxidative stress responses, including exposure to H_2_O_2_, were upregulated in Δ*mavR* compared to WT (Supplementary Figure S10A). RT-qPCR data were consistent with the RNAseq data as expression of *dps* (involved in oxidative stress and nutrient deprivation) and *poxB* (oxidative stress, metabolism) was increased in Δ*mavR* compared to WT, and these differences in expression were partially rescued by *mavR* complementation (Figure 7E). To test the biological significance of MavR in influencing EHEC survival following oxidative stress, we treated mid-logarithmic cultures of WT, Δ*mavR* and *mavR* complemented strains with 10 mM H_2_O_2_ and enumerated CFUs over time after exposure. At 1 h post-treatment, viable WT cells were at the limit of detection, whereas the Δ*mavR* strain was only slightly affected by the addition H_2_O_2_, which is consistent with the gene expression data. Furthermore, we were able to partially complement survival under H_2_O_2_ stress with p*mavR* (Supplementary Figure S10B). Collectively, these data suggest an extensive role for MavR in EHEC gene expression.

### MavR promotes expression of genes encoding flagella under aerobic conditions

**Figure 8.**
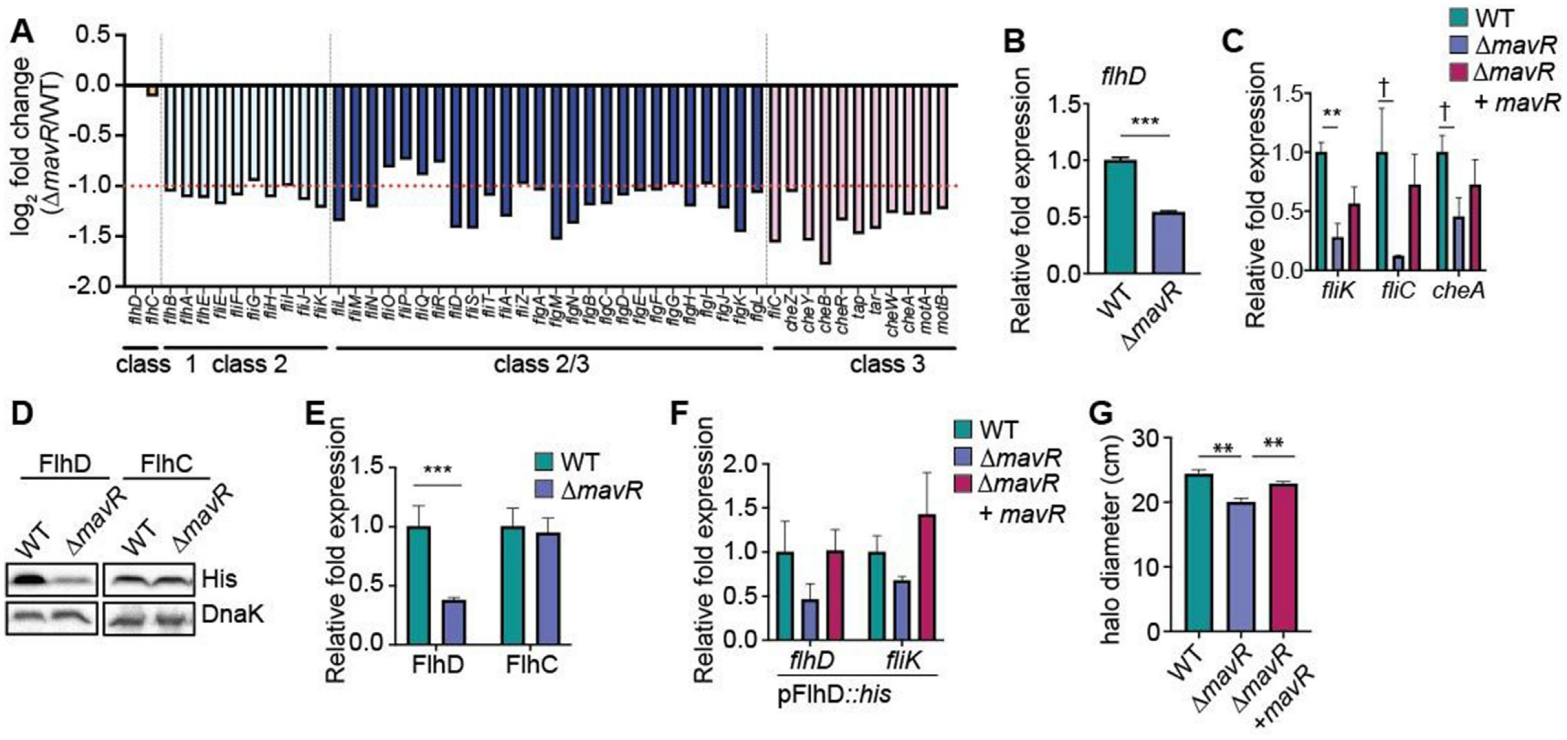
MavR promotes expression of genes encoding flagella. (**A**) RNAseq data showing flagella and motility gene expression in Δ*mavR* compared to WT grown aerobically. The dotted line indicates 2-fold change, *N* = 3. (**B**) RT-qPCR of *flhD* in WT and Δ*mavR*. (**C**) RT-qPCR of indicated genes identified as differentially expressed in the RNAseq data set in WT, Δ*mavR* and Δ*mavR* + pGEN-*mavR*. WT and Δ*mavR* carry the empty vector, *N* = 3. (**D**) Western blot of FlhD::His and FlhC::His in WT and Δ*mavR*. DnaK is the loading control. (**E**) Quantification of FlhD::His and FlhC::His expression in WT and Δ*mavR*,*N* = 3. (**F**) RT-qPCR of *flhD/flhD::his* and *fliK* in WT, Δ*mavR* and Δ*mavR* + pUCP24-*mavR*. WT and Δ*mavR* carry the empty vector, *N* = 3. (**G**) Quantification of WT, Δ*mavR*, and Δ*mavR*+ pGEN-*mavR* motility assays, *N* = 3. Bars represent the mean and error bars indicate SEM; ** *P* < 0.01, *** *P* < 0.001, † *P* < 0.1 (Student's two-sample *t*-test).

Notably, expression of nearly every gene that encodes flagellar biosynthesis or chemotaxis was decreased in Δ*mavR* compared to WT during aerobic growth (Figure 8A). We performed RT-qPCR to confirm differences in expression of *flhD*, *fliK*, *fliC*, and *cheA* (Figure 8B,C), which encode genes required for flagellar biosynthesis, motility, and chemotaxis. *Trans*-complementation with *mavR* expressed from the native promoter resulted in partial to nearly full complementation (Figure 8C). To determine whether MavR affected flagellar expression through EutR, we examined *flhD* and *fliK* expression in WT and Δ*eutR* grown without or with EA and AdoCbl supplementation. RT-qPCR analyses did not reveal a role for EutR in controlling expression of these genes. No significant differences in *flhD* or *fliK* expression were measured among any of the strains or growth conditions (Supplementary Figure S11A,B); however, EutR was required for EA/AdoCbl-dependent *eutS* expression (Supplementary Figure S11C). These data suggest that MavR influences expression of genes encoding flagella/ motility independent of EutR.

The heteromeric master regulator FlhDC controls transcription of genes encoding flagella and chemotaxis (83–85). Therefore, we examined whether MavR might affect flagella/chemotaxis gene expression via post-transcriptional control of FlhD and/or FlhC. For these experiments, we cloned each gene under the control of an arabinose-inducible promoter to remove native transcriptional regulation. FlhD expression was significantly decreased in Δ*mavR* compared to WT, whereas MavR did not influence FlhC expression (Figure 8D,E). To assess MavR modulation of *flhD* expression and the impact on FlhD target gene expression, we utilized the dual plasmid-based assay previously described. We measured a ∼2-fold decrease in *flhD* and *fliK* expression in Δ*mavR* compared to WT that was complemented by p*mavR* (Figure 8F). These data suggest that MavR promotes flagellar/chemotaxis gene expression by post-transcriptionally influencing expression of FlhD. Consistent with the gene expression data, Δ*mavR* was slightly less motile compared to WT, and this difference could be rescued upon complementation (Figure 8G). These results indicate that MavR post-transcriptionally promotes FlhD expression, which affects downstream genes.

### MavR promotes LEE expression and AE lesion formation

**Figure 9.**
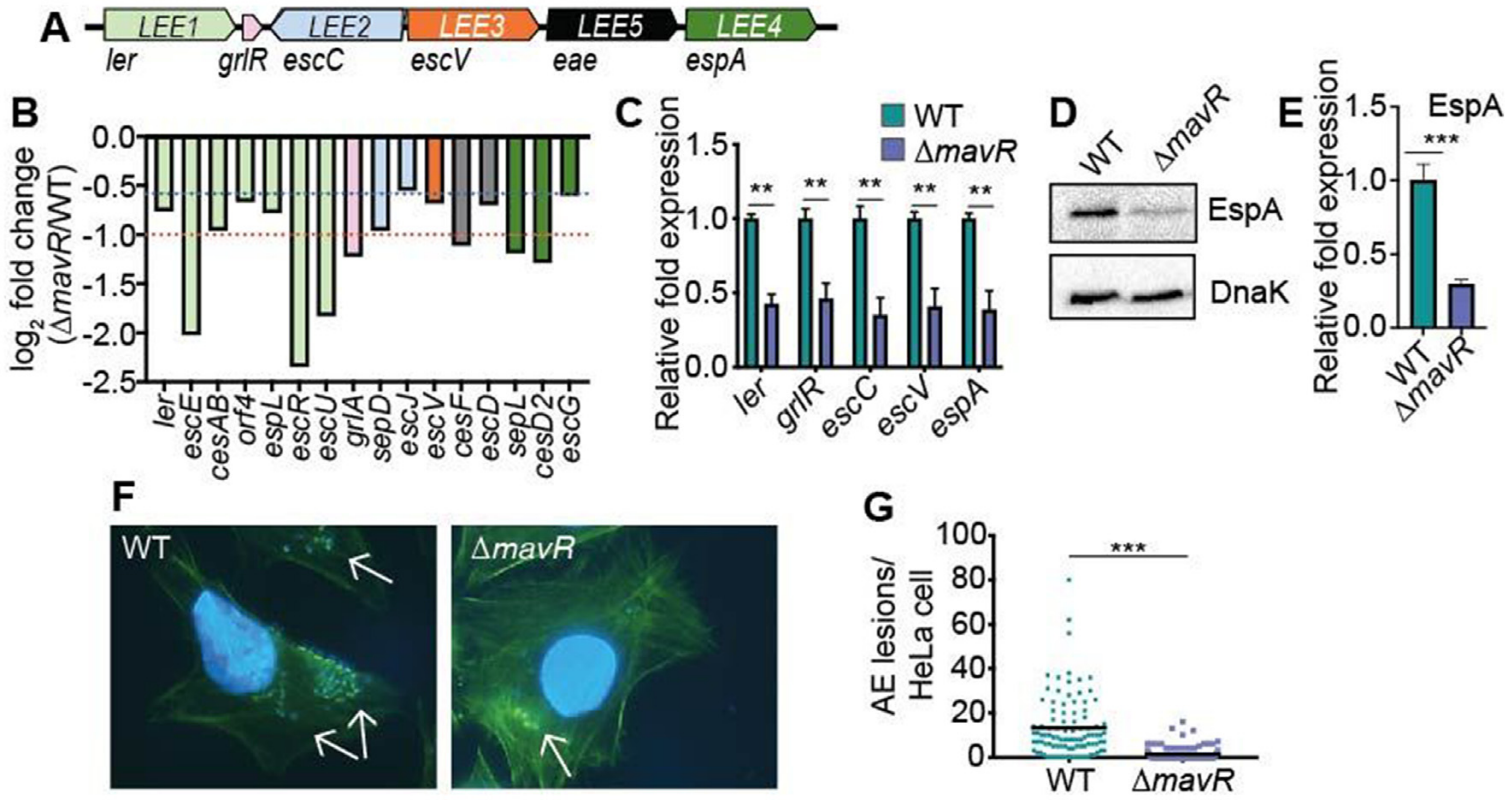
MavR is required for maximal LEE gene expression and AE lesion formation. (**A**) Schematic of the LEE. (**B**) RNAseq data comparing LEE gene expression in Δ*mavR* compared to WT under microaerobic conditions; *N* = 3. Columns are color-coded according to operon (shown in (A)). The red dotted line indicates 2-fold change, and the blue dotted line indicates 1.5-fold change. (**C**) RT-qPCR of LEE genes in WT and Δ*mavR*; *N* = 3. (**D**) Western blot of EspA expression in the WT and Δ*mavR*. DnaK is the loading control. (**E**) Quantification of EspA expression in the WT and Δ*mavR*. (**F**) FAS assay showing AE lesions on HeLa cells infected with WT or Δ*mavR*. AE lesions are indicated by arrows. (**G**) Quantification of AE lesions on HeLa cells infected with WT or Δ*mavR*. N = 98–120 HeLa cells. Bars represent the mean and error bars indicate SEM. ** *P* < 0.01, *** *P* < 0.001 (Student’s two-sample *t*-test).

The LEE pathogenicity island carries 41 genes that are mostly organized into five major operons (Figure 9A). The RNAseq data indicated that 16 LEE-encoded genes were at least 1.5-fold downregulated in Δ*mavR* compared to WT (Figure 9B). Importantly, expression of *ler*, that encodes Ler the master regulator of the LEE (11), was decreased 1.7-fold in Δ*mavR* (*P* = 0.079) (Figure 9B). We further analyzed LEE transcript levels by RT-qPCR. These data revealed at least 2-fold decreased LEE expression in Δ*mavR* vs WT, including decreased *ler* expression (Figure 9C). Moreover, western blot analysis confirmed that levels of EspA, which encodes the T3SS filament (encoded in *LEE4*) (86), were decreased in Δ*mavR* strain compared with WT EHEC (Figure 9D,E). Although overexpression of MavR (sRNA56) was previously reported to result in increased expression of *espA* (28), MavR overexpression did not complement the Δ*mavR* strain in these assays (data not shown). The LEE is required for the formation of attaching and effacing (AE) lesions on epithelial cells, and consistent with the gene expression data, Δ*mavR* formed significantly fewer AE lesions on HeLa cells compared to WT (Figure 9F,G). Together, these data reveal that MavR is required for robust LEE expression and demonstrate a role for MavR in EHEC virulence.

Bioinformatic queries did not predict MavR interaction with LEE transcripts and LEE transcripts were not enriched by MAPS, suggesting that MavR affects LEE expression indirectly. To date, over 40 transcription factors have been reported to control *ler* transcription (87–89). EutR directly activates *ler* expression (48,50). Additionally, the MAPS data indicated *phoB*, which encodes another transcriptional regulator of LEE expression (90), was a potential MavR target. We confirmed that MavR post-transcriptionally promoted PhoB expression (Supplementary Figure S12A,B) as well as PhoB-dependent gene expression (Supplementary Figure S12C). To test whether MavR and EutR- or PhoB-dependent LEE expression were functionally linked, we generated an *eutR* or *phoB* deletion in the Δ*mavR* background (generating Δ*mavR*Δ*eutR* and Δ*mavR*Δ*phoB* strains*)* and assessed EspA expression. Lower levels of EspA were detected in Δ*eutR* and Δ*phoB* compared to WT as previously reported. Notably, EspA levels were further decreased in Δ*mavR* and Δ*mavR*Δ*eutR*, as well as in Δ*mavR*Δ*phoB* (Supplementary Figure S12D-E). These results raise the possibility that MavR affects LEE expression by pleiotropic mechanisms.

### MavR is important for EHEC colonization of the mammalian GI tract

**Figure 10.**
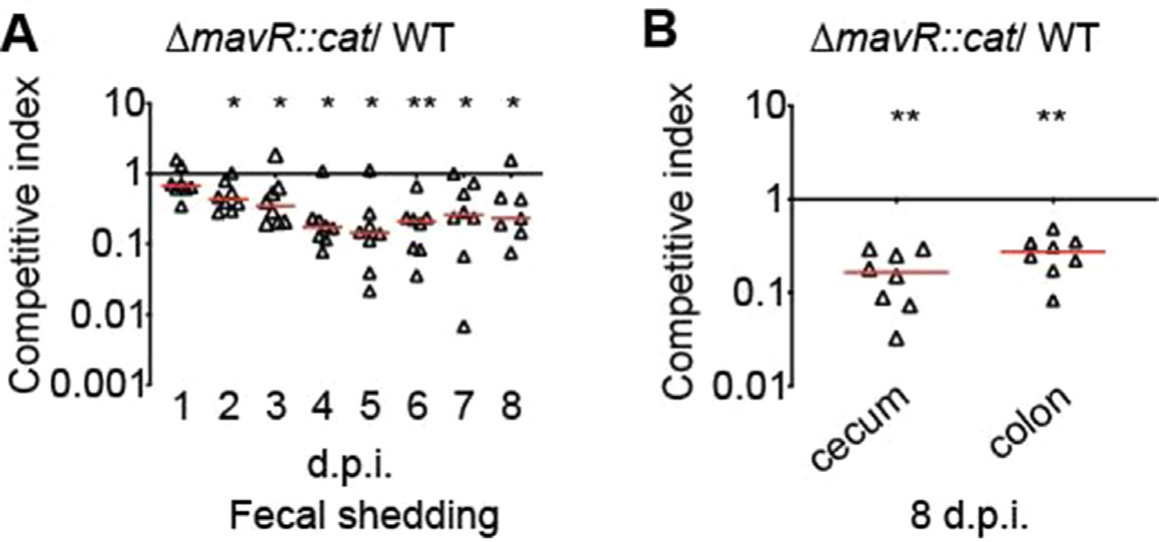
MavR is required for robust colonization of the GI tract. (**A**) Competition assay between WT and Δ*mavR::cat* strains harvested from fecal samples at indicated time points. (**B**) Competition assay between WT and Δ*mavR::cat* strains harvested from the cecum or colon. Each point represents a competitive index (CI). Horizontal lines represent the median value for each group. dpi, days post infection; *N* = 8. * *P* < 0.05, ** *P* < 0.01 (Wilcoxon log-rank test).

The extensive role for MavR in EHEC gene expression suggests MavR plays a critical role in EHEC adaptation to and fitness within the GI tract. Therefore, we performed competition experiments using streptomycin-treated mice. This model does not recapitulate LEE-dependent adherence to epithelial cells or AE lesion formation, but rather is used to evaluate the relative colonization capacity of an *E. coli* strain, including EHEC (91,92). Mice were orally infected with a 1:1 mixture of WT and Δ*mavR::cat* strains. At 2 days post infection and throughout the duration of the experiment, Δ*mavR::cat* was outcompeted by WT (∼10–100 fold) as reflected by CFUs in fecal samples (Figure 10A). These data were consistent with numbers of Δ*mavR::cat* and WT recovered from the cecum and colon (Figure 10B). These findings demonstrate that MavR is required for robust intestinal colonization.

## DISCUSSION

MavR was originally discovered via transcriptomic analyses to identify EHEC-specific and Hfq-dependent sRNAs. Initial characterization of MavR revealed that overexpression resulted in increased expression of the LEE-encoded gene *espA* (28), suggesting a role for MavR in virulence. Here, we employed two unbiased techniques, MAPS and RNAseq, to comprehensively elucidate the MavR regulon in EHEC grown under aerobic and microaerobic conditions. Although there was no overlap of putative targets/ differentially expressed genes among all of the data sets (Figure 7A), some genes were common in the aerobic datasets and microaerobic datasets, as well as transcripts that were enriched in both the aerobic and microaerobic MAPS. By performing these assays under distinct conditions, we determined that MavR affected expression of genes important for all phases of infection. Notably, previous studies have also demonstrated discrete sRNA–RNA interactions and regulatory outcomes depending on growth-phase (30) or growth medium (rich versus minimal/defined) (19,56). Collectively, these findings suggest that the regulon of a sRNA can vary under different growth and/or environmental conditions likely because the transcriptome—and therefore available RNA targets—varies under distinct conditions.

**Figure 11.**
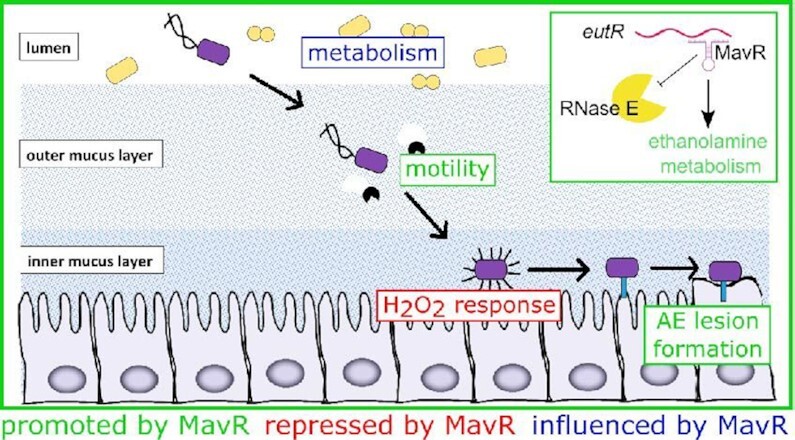
Summary of MavR-dependent gene expression in the context of EHEC host colonization. MavR affects expression of genes important for nutrient acquisition, motility, oxidative stress responses and AE lesion formation. The inset summarizes MavR-dependent activation of EutR expression and the impact on EA utilization.

The ability to acquire nutrients is an essential first step in host colonization and may enable a pathogen to overcome nutritional competition (43,46,93–95) (Figure 11). To overcome this challenge, EHEC utilizes diverse metabolic pathways to take advantage of a variety of metabolites (96). Our data reveal that MavR affected expression of genes important for biosynthesis and energy production, including EutR, the DNA-binding transcriptional activator of the *eut* locus. Subsequently, EHEC uses flagella to traverse the mucus layer and reach the epithelial border (97,98). There, EHEC encounters reactive oxygen species, including H_2_O_2_ (99,100). Finally, EHEC forms AE lesions which results in intimate attachment and nutrient acquisition (1,101,102). Our data indicate that MavR promoted expression of genes encoding flagella and the T3SS and repressed expression of genes important for oxidative stress responses (Figure 11). The concentration of H_2_O_2_ in the colonic lumen is sublethal (103,104). Therefore, it is possible that repression of oxidative stress response genes by MavR may reduce unnecessary energy expenditure to enhance survival in the GI tract. Finally, the *mavR* deletion strain was significantly attenuated during colonization of the mammalian GI tract (Figure 10), underscoring the importance of this sRNA to EHEC fitness during infection.

An important issue is understanding how MavR, and other sRNAs, coordinate expression of various targets. The competing endogenous RNAs (ceRNAs) hypothesis predicts that if a sRNA regulates several targets, an increase in the production of one target will cause sRNA availability for the other targets to decrease below the threshold value and result in deregulation (33,105,106). Maximal expression of EutR requires EA and AdoCbl (42,59). Interestingly, although *eutR* was identified as a putative MavR target by MAPS, subsequent targeted affinity purification experiments did not reliably reproduce these results (Figure 2). Notably, the affinity purification experiments were performed using medium lacking EA and AdoCbl, and subsequent experiments revealed that MavR affected *eutR* transcript levels only upon EA and AdoCbl supplementation (Figure 5) (or when native transcriptional control was removed [e.g. using an arabinose-inducible promoter]). Therefore, we hypothesize that MavR may be titrated away from less abundant targets, such as *eutR* in the absence of EA and AdoCbl, through competitive binding with more abundant transcripts. In addition to *eutR*, we identified a role for MavR in promoting PhoB expression. Similar to EutR, PhoB expression is responsive to environmental signals and occurs under phosphate starvation (107). A non-conflicting alternative hypothesis as to how MavR coordinates expression of diverse genes in EHEC may be due to MavR-dependent expression of transcription factors. Integration of transcriptional regulators into the regulatory network of an sRNA amplifies the regulon of the sRNA by adding indirect targets (108–110). Besides EutR and PhoB, MavR also post-transcriptionally promoted expression of the transcriptional regulator FlhD. Therefore, ceRNAs and/ or the incorporation of MavR into regulatory circuits may provide a mechanism for MavR to indirectly integrate discrete environmental signals to coordinate EHEC gene expression.

The ability to utilize EA as a carbon, nitrogen, and/or energy source is conserved among diverse bacteria. However, the complexity and organization of the *eut* genes varies greatly and can include only a few genes or many genes (111). The *Enterobacteriaceae* and the *Firmicutes* encode the longest *eut* operons, containing 17 and 19 genes, respectively. For decades, control of *eut* expression was thought to occur solely at the level of transcription initiation, via EutR, in the *Enterobacteriaceae* and the noncanonical two component system EutVW in the *Firmicutes* (112). More recently, several reports have characterized a complex mechanism in which a riboswitch-containing sRNA controls the activity of EutVW in *Enterococcus faecalis* and *Listeria monocytogenes* (*Firmicutes*) (63,64). Our data support a commonality in which expression of the energetically costly, long *eut* locus requires multiple layers of regulation and occurs at the level of the transcriptional activator. Expression of these long operons is only advantageous when both the metabolite EA and the required co-factor AdoCbl are present. In both systems, sRNAs positively regulate expression of these long operons only in the presence of both ligands.

The first characterized sRNAs were shown to interact with the 5′ untranslated region (5′ UTR) of the target mRNA. This resulted in sequestration or unmasking of the ribosome binding site (RBS) to inhibit or promote translation, respectively, or modulation of mRNA stability (20,113). Based on these original data, interactions with the 5′ UTR has become the canonical model of sRNA regulation. Notably, unbiased approaches to identify sRNA interacting partners are increasingly detecting interactions between sRNAs and the CDS of detected targets (e.g., (19,55,56); however, only a handful of studies have characterized the outcome of these interactions (including (24,34,36,114)). In the first example, the sRNA MicC was shown to promote target destabilization in *S. typhimurium* (36). Since then, additional examples have been reported in which a sRNA interacts with the CDS of its target transcript to repress expression (34,35). We previously reported that the sRNA DicF interacts with the CDS of the *pchA* transcript to promote translation (24). Subsequently, Chen *et al.* reported that the sRNA GcvB binds the *rbn* CDS to stabilize the transcript and promote expression (115). Our findings demonstrate that MavR also binds to the CDS to antagonize RNase E-dependent cleavage of the *eutR* transcript. Although the rifampicin experiments were not consistent, our model is supported by several lines of evidence. First, native *eutR* transcript levels were consistently decreased in Δ*mavR* compared to WT (Figure 5E, 6F, Supplementary Figure S7B). Second, the RNase E truncation rescued stability and expression of both tagged and native *eutR* in the Δ*mavR* strain compared to WT (Figure 6E–G). Finally, MavR protected the *eutR* transcript from RNase E mediated cleavage (Figure 6H). Thus, MavR-dependent regulation ensures maximal EutR expression and activation of *eut* expression and EA utilization (Figure 6I and 11). Thus, the data presented in this study expands upon the model that sRNAs promote gene expression through association with the CDS of a target transcript.

In summary, we provide the initial characterization of a novel sRNA, MavR, describe the global impact of MavR on EHEC gene expression, and present the functional consequences of MavR to fitness, motility, and virulence. We also report mechanistic insights as to how MavR promotes EutR expression. Further investigation is required to verify other potential targets identified in the MAPS experiments as well as to determine how MavR influences expression of these targets. Altogether, the findings presented herein reveal a striking role for a bacterial sRNA in niche adaptation and bacterial-host interactions.

## DATA AVAILABILITY

The sequencing data have been deposited in NCBI's Gene Expression Omnibus and are accessible through GEO Series accession number GSE166491 (https://www.ncbi.nlm.nih.gov/geo/query/acc.cgi?acc=GSE166491).

## Supplementary Material

gkab863_Supplemental_Files
