# Supplementary material for: A pathogen-specific sRNA influences enterohemorrhagic Escherichia coli fitness and virulence in part by direct interaction with the transcript encoding the ethanolamine utilization regulatory factor EutR
Source: Nucleic Acids Res. 2021 Sep 30;49(19):10988–1004. doi: 10.1093/nar/gkab863 (PMC8565329; doi:10.1093/nar/gkab863)
Supplement: gkab863_Supplemental_Files [file gkab863_supplemental_files.zip › 210823_SupplementalData.pdf]

A

-35 box -10 box TSS

TTGTCCGGATTGTAAA**TTCATA**TTAACTCTCCTGATTTTT**GATTATTAT**TAATGC**G**C

AGCGTTTATATATGTTCCCTAGGCTTAGTTCTGGACGCTGGATATTCGGTGAGGCGT

AAATATGGTATGACACCATTTTTCATAACGCTGAAGTTTCTATACCTGTTGAATTTG

AATTTTCATTGACCGGGTATCTTATTTTCCAGGGCCCGTTCCTTCATAAGTCGCAAA

AGTAACATATATCCGAAGGGCATGCTGTTGATATCAGACACGGAATACTGGCTTTAA

CCAGCAACCACAGATAAACCCGGGGCCTGCATAAAAGGTTCATGCCAGAATTAACAT

AGCTCTGCTTTCTGCCCCCCCCTTTCCCATATTCACCCGTTGATAGCGGATCGATAC

CCAAAAAAAACCCCGGCATTGCCGGGGTCAGACTCAGC

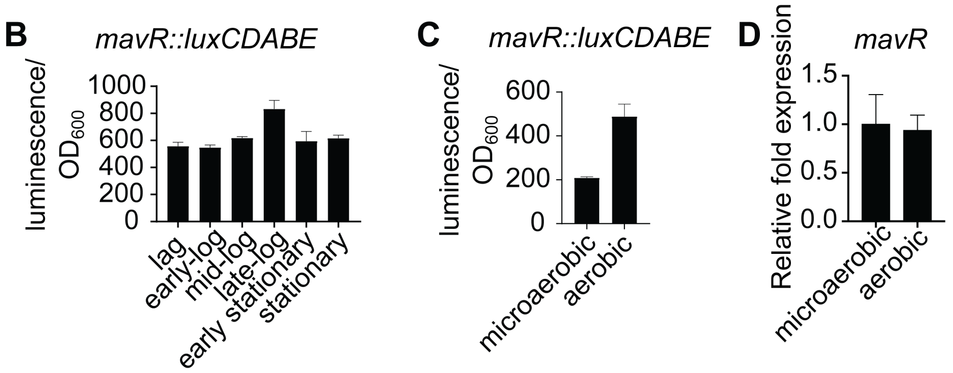

Fig. S1. A) Sequence of *mavR* with genomic features annotated. B) & C) Expression of *luxCDABE* from the MavR promoter normalized to O.D.$_{600}$ B) at different growth phases (aerobic growth) or C) under microaerobic and aerobic conditions. D) RT-qPCR of *mavR* expression in EHEC grown under microaerobic or aerobic conditions. For all, bars represent the mean and error bars indicate SEM. N=3.

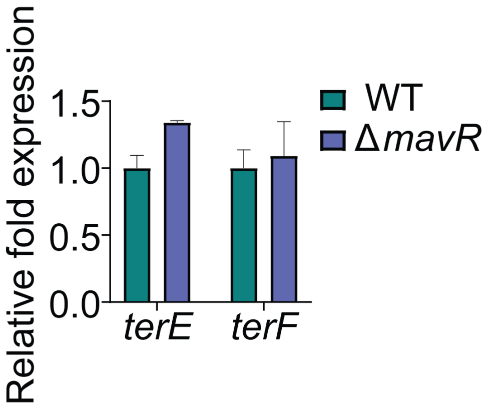

Fig. S2. RT-qPCR of *terE* and *terF* expression in WT and Δ*mavR*. Bars represent the mean and error bars indicate SEM. N=3.

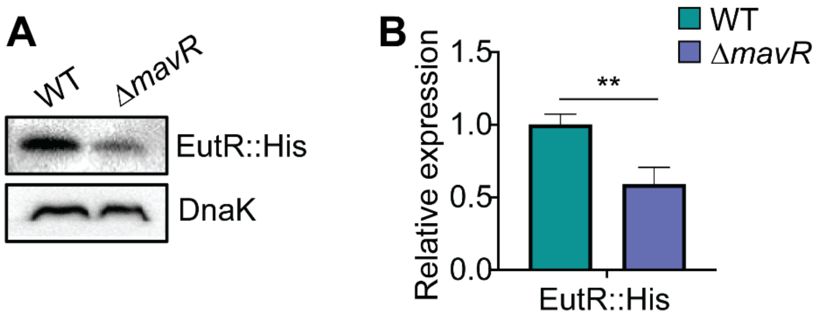

Fig. S3. A) Western blot of EutR::His in WT and Δ*mavR.* DnaK is the loading control. B) Quantification of EutR::His expression in WT and Δ*mavR*. N=9. ** $p<0.01$ (student's two-sample t-test).

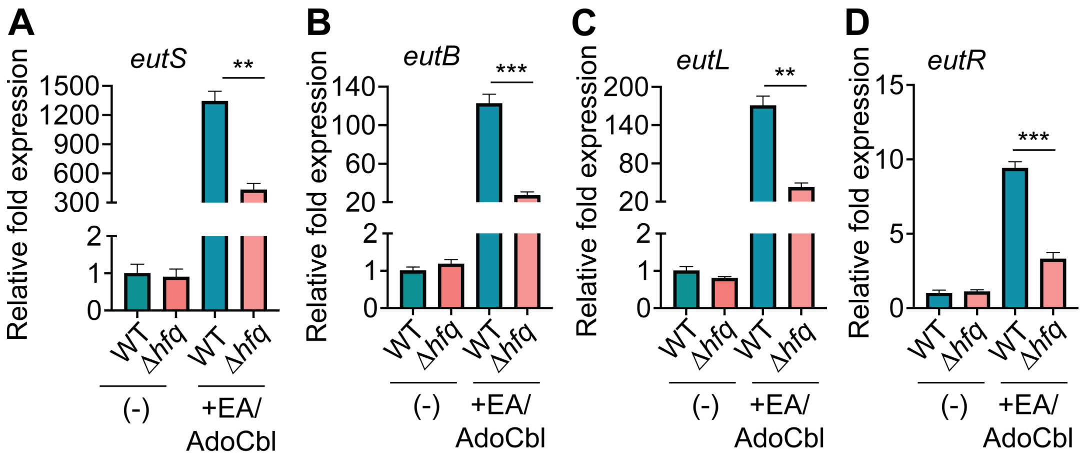

Fig. S4. RT-qPCR of *eut* gene expression in WT and Δ*hfq*. Bars represent the mean and error bars indicate SEM. N=3. ** p<0.01, *** p <0.001 (student's two-sample t-test).

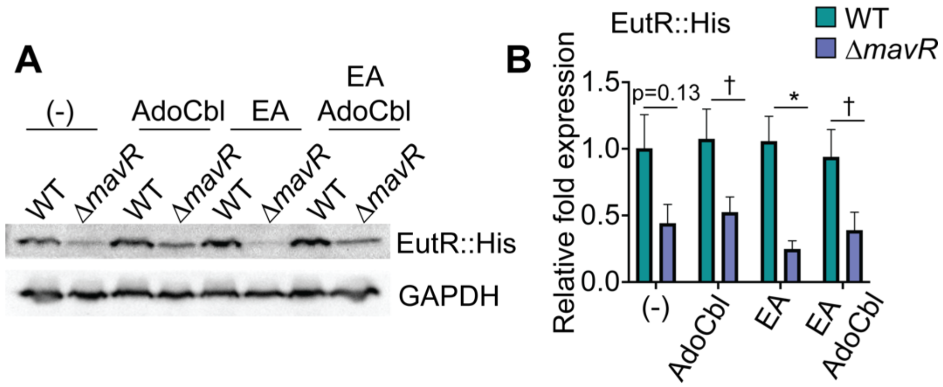

Fig. S5. A) Western blot of EutR::His. GAPDH is a loading control. B) Quantification of EutR::His in WT and Δ*mavR*. Bars represent the mean and error bars indicate SEM. N=3. * $p<0.05$, † <0.1 (student's two-sample t-test).

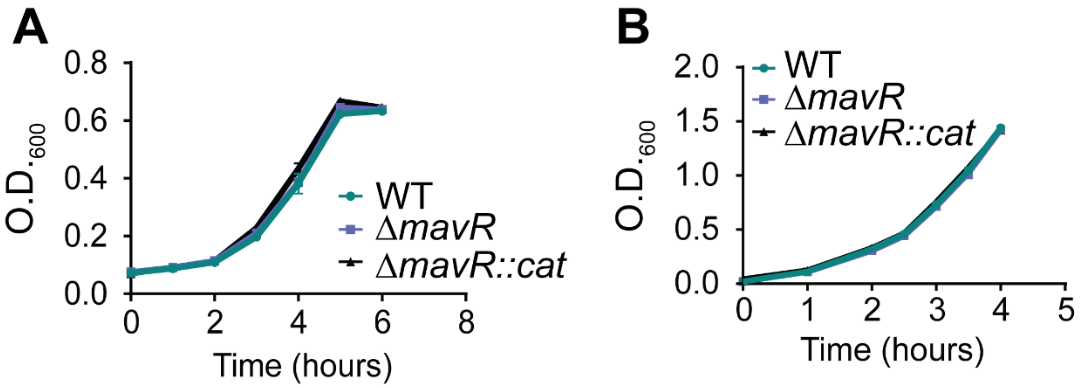

Fig. S6. Growth curves of WT, Δ*mavR*, and Δ*mavR::cat* strains under A) microaerobic or B) aerobic conditions in DMEM. Error bars indicate SEM. N=3.

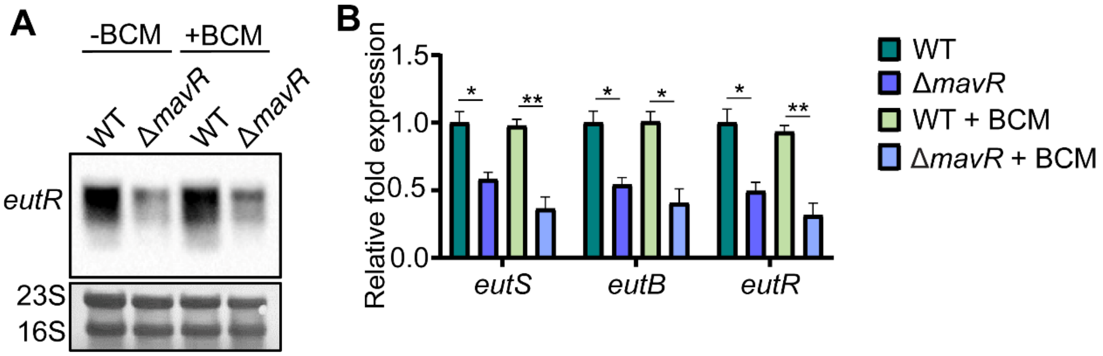

Fig. S7. A) Northern blot of *eutR::His* from RNA extracted from cultures treated with vehicle or BCM. 23S and 16S rRNA are loading controls. B) RT-qPCR of *eutS*, *eutB*, and *eutR* using RNA extracted from cultures treated with vehicle or BCM. * $p<0.05$, ** $p<0.01$ (student's two-sample t-test).

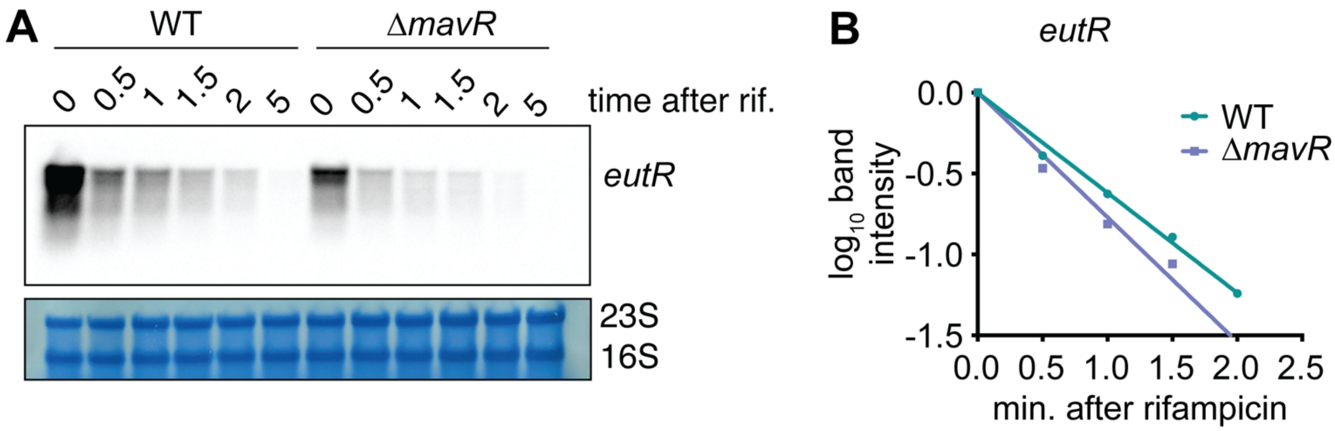

Fig. S8. A) Northern blot of *eutR* transcripts in WT and Δ*mavR* at indicated time points before or after addition of rifampicin. 16S and 23S rRNA are the loading controls. B) RNA decay curves of *eutR* transcript in WT and Δ*mavR*. The signal obtained at 0 min. was set to 1 for each strain, and the amount of RNA remaining at each timepoint was plotted on the *y*-axis vs time on the *x*-axis. N=2. In A) and B), WT and Δ*mavR* were transformed with pBAD::*eutR*.

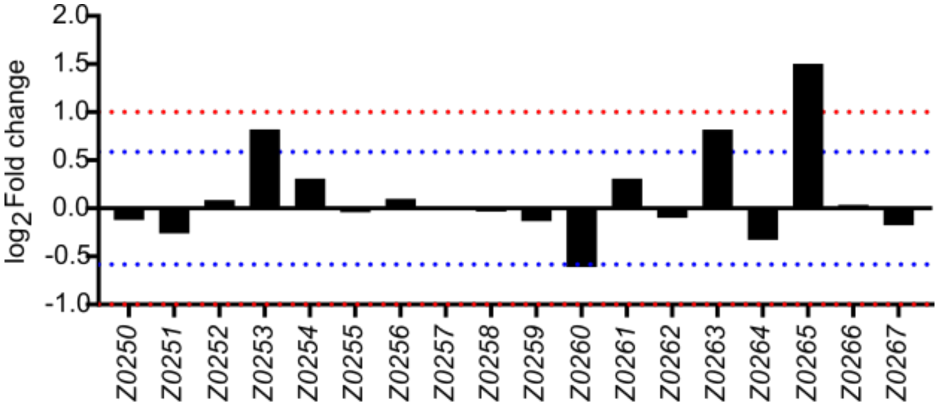

Fig. S9. RNAseq data showing differentially expressed transcripts encoding T6SS proteins in WT and Δ*mavR*. N=3. The red dotted line indicates 2-fold change, and the blue dotted line indicates 1.5-fold change.

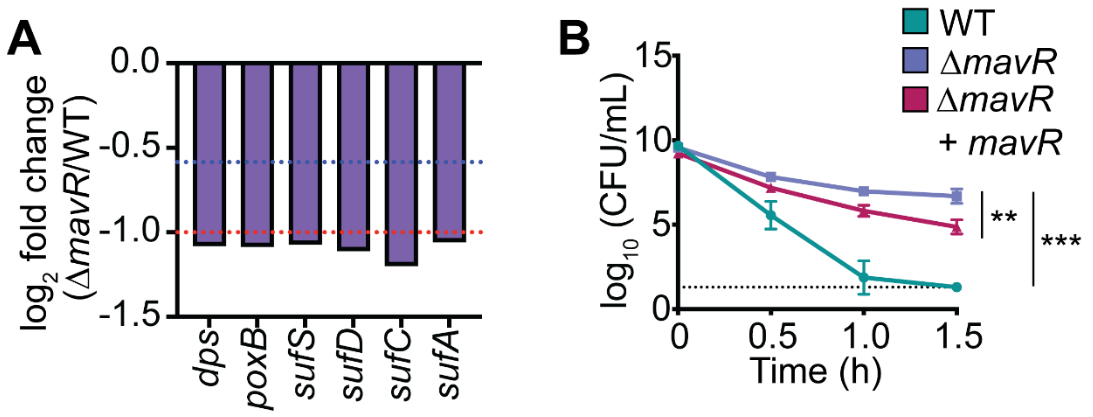

Fig. S10. A) RNAseq data comparing gene expression in Δ*mavR* compared to WT under microaerobic conditions. N=3. The red dotted line indicates 2-fold change, and the blue dotted line indicates 1.5-fold change. B) Viability of WT, Δ*mavR,* and Δ*mavR* + pBAD-*mavR*::His after exposure to 10 mM H_2O_2. The dashed line represents the limit of detection. N=3. Bars or symbols represent the mean and error bars indicate SEM. * $p<0.05$, ** $p<0.01$, *** $p <0.001$ (student's two-sample t-test).

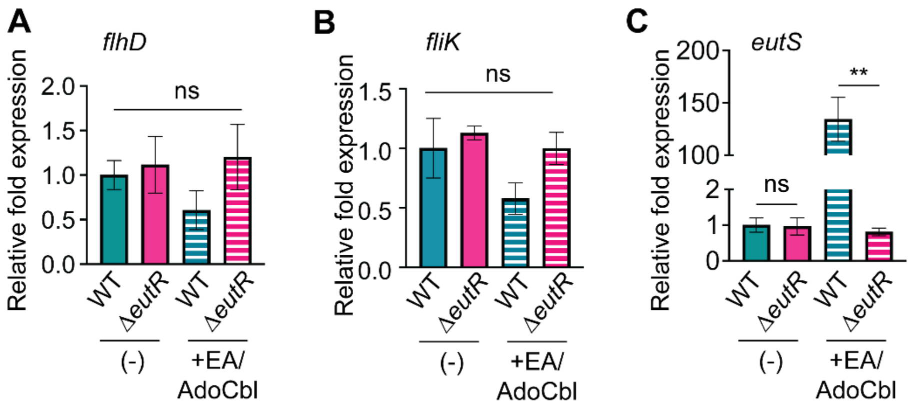

Fig. S11. RT-qPCR of A) *flhD;* B) *flik;* and C) *eutS* gene expression in WT and Δ*eutR* grown without or with ethanolamine (EA) and adenosylcobalamin (AdoCbl) supplementation. Bars represent the mean and error bars indicate SEM. N=3. ns p>0.05, ** p<0.01 (student's two-sample t-test).

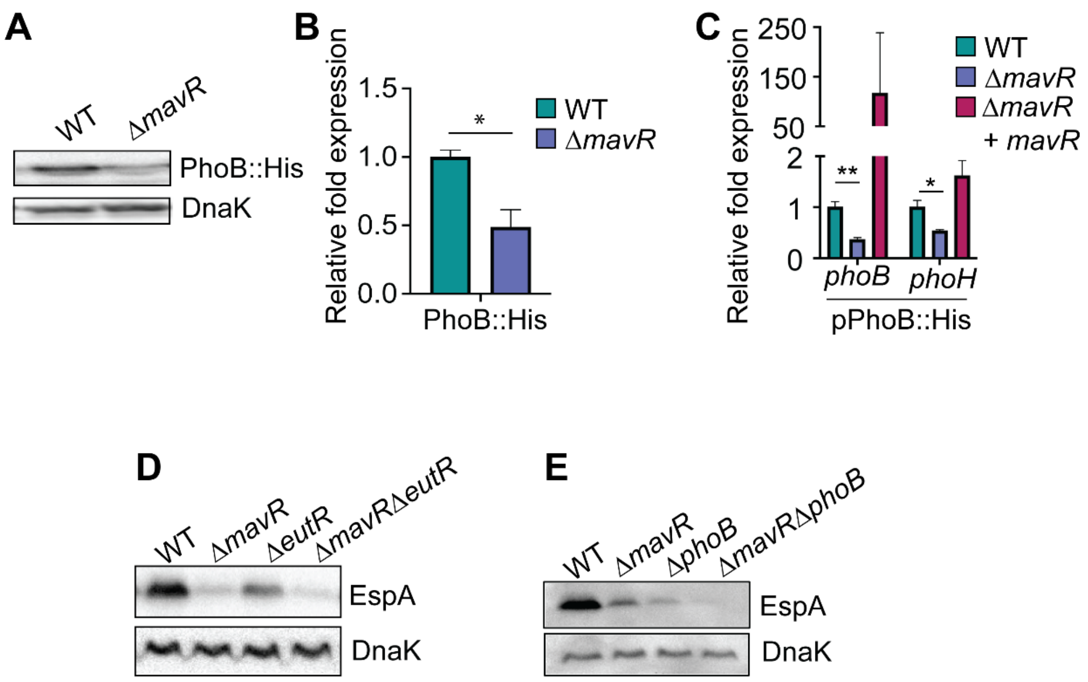

Fig. S12. A) Western blot of PhoB::His in WT and Δ*mavR.* DnaK is the loading control. B) Quantification of PhoB::His expression in WT and Δ*mavR*. N=3. C) RT-qPCR of *phoB/phoB::his* and *phoH* in WT, Δ*mavR,* and Δ*mavR* + pUCP24-*mavR*. WT and Δ*mavR* carry the empty vector. N=3. D) Western blot of EspA expression in WT, Δ*mavR*, Δ*eutR,* and Δ*mavR*Δ*eutR*. DnaK is the loading control. E) Western blot of EspA expression in the WT, Δ*mavR*, Δ*phoB,* and Δ*mavR*Δ*phoB*. DnaK is the loading control. * $p<0.05$, ** $p<0.01$ (student's two-sample t-test).

Table S1. Strains used in this study.

**Strain**	**Description**	**Reference**
86-24	WT EHEC O157:H7	(*1*)
mavR::*cat*	86-24 with *mavR* replaced with *cat*	This study
Δ*mavR*	86-24 *mavR* deletion strain	This study
Δ*hfq*	86-24 *hfq* deletion strain	(*2*)
rne$^{\Delta CTD}$	86-24 with truncated *rne* at residue 426	This study
Δ*mavR rne*$^{\Delta CTD}$	86-24 *mavR* deletion strain and truncated *rne* at residue 426	This study
Δ*eutR*	86-24 *eutR* deletion strain	(*3*)
Δ*mavR*Δ*eutR*	86-24 *mavR* and *eutR* deletion strain	This study
Δ*phoB*	86-24 *phoB* deletion strain	This study
Δ*mavR*Δ*phoB*	86-24 *mavR* and *phoB* deletion strain	This study

Table S2. Plasmids used in this study.

**Plasmid**	**Description**	**Reference**
pKD3	pANTSλ derivative containing FRT-flanked chloramphenicol resistance	(*4*) Addgene 45604
pKD4	pANTSλ derivative containing FRT-flanked kanamycin resistance	(*4*) Addgene 45605
pKD46	Lambda red recombinase expression plasmid	(*4*) CGSC 7739
pCP20	FLP recombinase expression plasmid with temperature sensitive origin of replication	(*4*) CGSC 14177
pBAD/mychis A	Expression vector containing arabinose inducible promoter and Myc/His tag	Thermo V44001
pBAD-*eutR*	pBAD/mychis A containing *eutR* inserted with KpnI and HindIII	This study
pBAD-*phoB*	pBAD/mychis A containing *phoB* inserted with KpnI and HindIII	This study
pBAD-*flhD*	pBAD/mychis A containing *flhD* inserted with KpnI and HindIII	This study
pBAD-*flhC*	pBAD/mychis A containing *flhC* inserted with KpnI and HindIII	This study
pBAD-*mavR*	pBAD/mychis A containing *mavR* inserted with KpnI and HindIII	This study
pBAD-N-rne	pBAD/mychis A containing N-rne inserted with KpnI and HindIII	This study
pHMM	Expression vector containing MBP/hexahistidine tagged MS2 coat protein	(*5*) Addgene 67717
pBAD-MS2-*mavR*	pBAD-*mavR* containing the MS2 aptamer	This study
pBAD-MS2	pBAD-MS2-*mavR* with *mavR* deleted	This study
pUCP24	Expression vector containing IPTG inducible promoter	(*6*)
pUC-*mavR*	pUCP24 containing *mavR*	This study

pBAD24	Expression vector containing arabinose inducible promoter	(*7*)
pBAD24-eutR	pBAD24 containing eutR inserted with KpnI and HindIII	This study
pGEN-MCS	Vector for expression from native promoter	(*8*) Addgene 44919
pGEN-mavR	pGEN-MCS containing *mavR* inserted with HindIII and NheI	This study
pGEN-luxCDABE	synthetic M7 promoter, constitutive lux expression	(*8*) Addgene 44918
plux-*mavR*	pGEN-luxCDABE with M7 promoter replaced with *mavR* promoter	This study

Table S3. Oligonucleotides used in this study. Lambda Red sequences are bolded and restriction enzyme sites are underlined.

Purpose	Primer name	Sequence
**Lambda Red**		
Delete *mavR*	*mavR*_LR_fwd	CTCTCCTGATTTTTGATTATTATTAATGCGCAGCGTTTATATATGTTCCCT**GTGTAGGCTGGAGCTGCTTC**
	*mavR*_LR_rev	AAAGCAGAGCTATGTTAATTCTGGCATGAACCTTTTATGCAGGCCCCGGGT**CATATGAATATCCTCCTTAG**
Sequence *mavR*	*mavR* _up	TTTACTGACGTTCGCCGGAA
	*mavR*_down	TGTCTGAAGACGCCTCAACC
Delete CTD of *rne*	Rne_LR_fwd	CTGGCACCGTGCGTGACAACGAATCGCTGTCGCTCTCTATTCTGCGTCTG**GTGTAGGCTGGAGCTGCTTC**
	Rne_LR_rev	TTACTCAACAGGTTGCGGACGCGCAGGAGCGGCAGAGGCATGATGCGTTG**CATATGAATATCCTCCTTAG**
Sequence *rne*	Rne_up	ACCGTGCGCGTATTCAAATCA
	Rne_down	CGATGAATTTTAATATGTTGATT
Delete *phoB*	phoB_LR_fwd	ATTTATTACAACAGGGCAAATCATGGCGAGACGTATTCTG**GTGTAGGCTGGAGCTGCTTC**
	phoB_LR_rev	CCTGCTCTGCGTCCGATGAGCAAGGCGTTAAAAGCGGGTT**CATATGAATATCCTCCTTAG**
Sequence *phoB*	phoB_up	ATGTGCGACGAGCTTTTCAT
	phoB_down	GATGCCAGCAAAAACCAGGG
**Vectors**		
Generate pBAD-*eutR*	eutR_pBmh_fwd	ATCGGTACCCCTGTCGGAGGTGCCGGG
	eutR_pBmh_rev	ATCAAGCTTCCCCCATTCCCGCATCCG
Mutate 11bp in pBAD-*eutR*	eutR_mut_fwd	CAATCCCCCAAGCTTGGGCCCGAACA
	eutR_mut_rev	GGGCATGGCCTGATGCAACGTCAACGAC
Mutate 3 RNase E motifs	eutR_mutrne3_fwd	AATACGCCACGGACTACCAGCAGCTGTTTGCCG
	eutR_mutrne3_rev	GCCCCAGATGCCAGTATCCCCACTGCATGGCGG
Generate pBAD-*flhC*	flhC_pBmh_fwd	ACGGTACCGAAAAGGGCCTGATCATGAG
	flhC_pBmh_rev	ACAAGCTTAACAGCCTGTACTCTCTGT
Generate pBAD-*flhD*	flhD_pBmh_fwd	ACGGTACCAGTACAGTTGCGTCGATTT
	flhD_pBmh_rev	ACAAGCTTGGCCCTTTTCTTGCGCAG
Generate pBAD-*phoB*	phoB_pBmh_Fwd	ACGGTACCCGCAACCTATTTATTACAACAG
	phoB_pBmh_Rev	ACAAGCTTAAAGCGGGTTGAAAAACG
	*mavR*_comp_fwd	ATCGGTACCGCAGCGTTTATATATGTTCC

Generate pBAD-*mavR* and pUC-*mavR*	*mavR*_comp_rev	ATCAAGCTTCTAAAAGACGCTGAGTCTGA
Generate pBAD-MS2*mavR*	pBAD_MS2*mavR*_fwd	AGGTACCCGTACACCATCAGGGTACGTTTTTCGTACACCATCAGGGTACG***G***CAGCGTTTATATATGTTCC
Generate pBAD-MS2	MS2_del*mavR*_fwd	AAGCTTGGGCCCGAACAA
	MS2_del*mavR*_rev	CGTACCCTGATGGTGTAC
Generate pGEN-*mavR*	mavR_pGEN_fwd	ATCGAAGCTTTTTACTGACGTTCGCCGGAA
	mavR_pGEN_rev	ATCGGCTAGCTGTCTGAAGACGCCTCAACC
Generate plux-*mavR*	*mavR*_plux_fwd	ACGTTTAAACTCGTAGATTGCTGAAGGGGT
	*mavR*_plux_rev	ACTACGTACTAGGGAACATATATAAACGCTG
Generate pBAD-N-rne-mychis	N-rne_pBmh_Fwd	ACGGTACCAAAAGAATGTTAATCAACGCAACTCAGC
	N-rne_pBmh_Rev	ACAACGTTAGCGCAGGTTGTTCCGGAC
Sequence pUCP24	M13_fwd	TGTAAAACGACGGCCAGT
	M13_Rev	CAGGAAACAGCTATGAC
Sequence pBAD	pBAD_fwd	ATGCCATAGCATTTTTATCC
	pBAD_rev	TGATTTAATCTGTATCAGG
Sequence plux	pGEN_fwd	GGCACTTGCTCACGCTCTG
	pGEN_rev	GTGGTCACGCTTTTCGTTGG
***In vitro* transcription**		
9S EMSA probe	9S_EMSA_fwd	TAATACGACTCACTATAGGAAGGTGTTTTGGCGGAT
	9S_EMSA_rev	AACGCAAAAAGGCCATCCTGAC
eutR EMSA probe	eutR_EMSA_fwd	TAATACGACTCACTATAGAGGAATTGGCCCGAACGCGTG
	eutR_EMSA_rev	TCACCCCCATTCCCGCATCCG
mavR EMSA probe	*mavR*_EMSA_fwd	TAATACGACTCACTATAGGCAGCGTTTATATATGTTCCCTAGGCTTAG
	*mavR*_EMSA_rev	GTCTAAAAGACGCTGAGTCTGA
100 bp *mavR*	mavR_100bp_fwd	TAATACGACTCACTATAGAACCAGCAACCACAGATAAAC
100 bp *eutR*	eutR_100bp_fwd	TAATACGACTCACTATAGGCCATGCAGTGGGGATTCTGG
700 bp *eutR*	eutR_700bp_fwd	TAATACGACTCACTATAGAGAAATAGCGACGCGCCC
mavR NB probe	*mavR*_NB_fwd	GGCTTAGTTCTGGACGC
	*mavR*_NB_rev	TAATACGACTCACTATAGGGCAGTATTCCGTGTCTGATATCAAC
eutR NB probe	eutR_NB_fwd	TGTGCTGGAAAACATGTCCGAA
	eutR_NB_rev	TAATACGACTCACTATAGCTTTGCGACCACGGGCTTAT
**RT-qPCR**		
	adhP_qRT_fwd	GTCACCACCTACAAAGCCGT
	adhP_qRT_rev	CCGCCAAGACCGTAGATAGC
	cheA_qRT_fwd	AACTGACTGCGACTCTGCTC
	cheA_qRT_rev	GGCATTAGAAGCGAAAGGCG
	comR_qRT_fwd	GTCACAGTGGGTCGGGATTT
	comR_qRT_rev	CCCTCTCCCCAGATTCAGGA
	dnaK_qRT_fwd	ATGCTGACGGTATCCTGCAC
	dnaK_qRT_rev	CCAGAAGACGCCTTGATGGT
	dps_qRT_fwd	CCCGCAACGATGTCTCTGAC
	dps_qRT_rev	TCCAGTGCGCTTGTTTGGTA
	eae_qRT_fwd	GCTGGCCCTTGGTTTGATCA
	eae_qRT_rev	GCGGAGATGACTTCAGCACTT
	elaB_qRT_fwd	CTGAAAGCGCGTGCAGAAAA

	elaB_qRT_rev	CCTGCTTCGCCCGATAGTAA
	eno_qRT_fwd	TGGCGCGAAAACTGTGAAAG
	eno_qRT_rev	TAGCCACCTTCGTCACCAAC
	escC_qRT_fwd	GCGTAAACTGGTCCGGTACGT
	escC_qRT_rev	TGCGGGTAGAGCTTTAAAGGCAAT
	escV_qRT_fwd	TCGCCCCGTCCATTGA
	escV_qRT_rev	CGCTCCCGAGTGCAAAA
	espA_qRT_fwd	TCAGAATCGCAGCCTGAAAA
	espA_qRT_rev	CGAAGGATGAGGTGGTTAAGCT
	evgS_qRT_fwd	TGAAGTCACAGCAGAGACGAA
	evgS_qRT_rev	ATAAACAGCATGTTCGCTGGC
	eutS_ qRT_fwd	TTATGACGCTAACTCCCGGC
	eutS_qRT _rev	CCACTGAAGCGGTCGAGAAA
	eutB_qRT_fwd	CGGCATCCCCATGATGTAGT
	eutB_qRT_rev	ACCTCAACGAAAACCTGATGATC
	eutL_qRT_fwd	CCGCCACATATTCGTAGCCT
	eutL_qRT_rev	CATCGCTTGTTTGGTCGCTT
	eutR_qRT_fwd	TTGCCCCAGATGCCAGAA
	eutR_qRT_rev	CGCAAAGCACAACGGTAAAAG
	flhD_qRT_fwd	TTTCGTCTCGGCATAAATGAAG
	flhD_qRT_rev	TCATTCAGCAAGCGTGTTGAG
	fliC_qRT_fwd	CCCTTCATGCTGATGTGGGT
	fliC_qRT_rev	TCAAGTTGCCTGCATCGTCT
	fliK_qRT_fwd	CAGCGATATTGTTTCCGACGC
	fliK_qRT_rev	CATCGTCTGAGCGGTGGTTA
	grlR_qRT_fwd	AAGACTCCTGTGGGGAAGGT
	grlR_qRT_rev	AGGACCCCCTGATACACAGA
	ler_qRT_fwd	CGACCAGGTCTGCCC
	ler_qRT_rev	GCGCGGAACTCATC
	mavR5'_qRT_fwd	CAGGGCCCGTTCCTTCATAA
	mavR5'_qRT_rev	CCGGGTTTATCTGTGGTTGC
	mavR3'_qRT_fwd	CTGGATATTCGGTGAGGCGT
	mavR3'_qRT_rev	GGAACGGGCCCTGGAAAATA
	phoH_qRT_fwd	TCTCCTCCGGTAGTCATCTCG
	phoH_qRT_rev	CAGAGGGTTGGCTTCCTTGT
	phoB_qRT_fwd	TTGCCGGGTGATGTATCTCG
	phoB_qRT_rev	GACCACTCGCGTGTCAAAAC
	poxB_qRT_fwd	GGGCGATTTCCTCTCGGTAG
	poxB_qRT_rev	GCTCAGTACCGTCTGTCAGG
	rpoA_qRT_fwd	GCGCTCATCTTCTTCCGAAT
	rpoA_qRT_rev	CGCGGTCGTGGTTATGTG
	terE_qRT_fwd	GCATGGTGAGCAACAGCTTC
	terE_qRT_rev	CATTGCGGTTTCGGTTGAGG
	terF_qRT_fwd	AAAGGCGAACCCGATACCTG
	terF_qRT_rev	GAACCGGTAACGAGCCTGAC
	tnaA_qRT_fwd	TGTACACCGAGTGCAGAACC
	tnaA_qRT_rev	CCGTCATACAGACCTACGGC
	wzzB_qRT_fwd	GCGTCACATCTTCAGTCTGC
	wzzB_qRT_rev	CAGGAAGTAGTTGCGCAGGA

	QT_raceadapter	CCAGTGAGCAGAGTGACGAGGACTCGAGCTCAAGCTTTTTTTT TTTTTTTTT
	QO_RACEout	CCAGTGAGCAGAGTGACG
	QI_racein	GAGGACTCGAGCTCAAGC

Table S4. Bioinformatic programs used in this study.

Program	URL	References
Bowtie2	http://bowtie-bio.sourceforge.net/bowtie2/index.shtml	(*9*, *10*)
Bprom	http://www.softberry.com/berry.phtml?topic=bprom&group=programs&subgroup=gfindb	(*11*)
CopraRNA	http://rna.informatik.uni-freiburg.de/CopraRNA/Input.jsp	(*12*-*14*)
DAVID	https://david.ncifcrf.gov/summary.jsp	(*15*, *16*)
Deseq2	http://bioconductor.org/packages/release/bioc/html/DESeq2.html	(*17*)
Graphpad Prism	https://www.graphpad.com/scientific-software/prism/	commercial
Hilighter	https://www.hiv.lanl.gov/content/sequence/HIGHLIGHT/highlighter_top.html	(*18*)
Htseq	https://htseq.readthedocs.io/en/master/	(*19*)
IntaRNA	http://rna.informatik.uni-freiburg.de/IntaRNA/Input.jsp	(*12*, *14*, *20*, *21*)
TargetRNA2	http://cs.wellesley.edu/~btjaden/TargetRNA2/	(*22*)

Supplementary References

1. P. M. Griffin *et al.*, Illnesses associated with *Escherichia coli* O157:H7. *Ann. Intern. Med.* **109**, 705-712 (1988).
2. M. M. Kendall, C. C. Gruber, D. A. Rasko, D. T. Hughes, V. Sperandio, Hfq virulence regulation in enterohemorrhagic Escherichia coli O157:H7 strain 86-24. *J Bacteriol* **193**, 6843-6851 (2011).
3. M. M. Kendall, C. C. Gruber, C. T. Parker, V. Sperandio, Ethanolamine controls expression of genes encoding components involved in interkingdom signaling and virulence in enterohemorrhagic Escherichia coli O157:H7. *MBio* **3**, (2012).
4. K. A. Datsenko, B. L. Wanner, One-step inactivation of chromosomal genes in Escherichia coli K-12 using PCR products. *Proc Natl Acad Sci U S A* **97**, 6640-6645 (2000).
5. R. Batey, J. Kieft, Improved native affinity purification of RNA. *RNA (New York, N.Y.)* **13**, (2007).
6. S. West, H. Schweizer, C. Dall, A. Sample, L. Runyen-Janecky, Construction of improved Escherichia-Pseudomonas shuttle vectors derived from pUC18/19 and sequence of the region required for their replication in Pseudomonas aeruginosa. *Gene* **148**, (1994).
7. L. Guzman, D. Belin, M. Carson, J. Beckwith, Tight regulation, modulation, and high-level expression by vectors containing the arabinose PBAD promoter. *Journal of bacteriology* **177**, (1995).
8. M. Lane, C. Alteri, S. Smith, H. Mobley, Expression of flagella is coincident with uropathogenic Escherichia coli ascension to the upper urinary tract. *Proceedings of the National Academy of Sciences of the United States of America* **104**, (2007).
9. B. Langmead, S. Salzberg, Fast gapped-read alignment with Bowtie 2. *Nature methods* **9**, (2012).
10. B. Langmead, C. Wilks, V. Antonescu, R. Charles, Scaling read aligners to hundreds of threads on general-purpose processors. *Bioinformatics (Oxford, England)* **35**, (2019).

11. V. Solovyev, A. Salamov, in *Metagenomics and its Applications in Agriculture, Biomedicine and Environmental Studies*. (Nova Science Publishers, 2011), pp. 61-78.
12. M. Raden *et al.*, Freiburg RNA tools: a central online resource for RNA-focused research and teaching. *Nucleic Acids Res* **46**, W25-w29 (2018).
13. P. Wright *et al.*, Comparative Genomics Boosts Target Prediction for Bacterial Small RNAs. *Proceedings of the National Academy of Sciences of the United States of America* **110**, (2013).
14. P. R. Wright *et al.*, CopraRNA and IntaRNA: predicting small RNA targets, networks and interaction domains. *Nucleic Acids Res* **42**, W119-123 (2014).
15. D. Huang, B. Sherman, R. Lempicki, Bioinformatics enrichment tools: paths toward the comprehensive functional analysis of large gene lists. *Nucleic acids research* **37**, (2009).
16. D. Huang, B. Sherman, R. Lempicki, Systematic and integrative analysis of large gene lists using DAVID bioinformatics resources. *Nature protocols* **4**, (2009).
17. M. Love, W. Huber, S. Anders, Moderated estimation of fold change and dispersion for RNA-seq data with DESeq2. *Genome biology* **15**, (2014).
18. B. Keele *et al.*, Identification and characterization of transmitted and early founder virus envelopes in primary HIV-1 infection. *Proceedings of the National Academy of Sciences of the United States of America* **105**, (2008).
19. S. Anders, P. Pyl, W. Huber, HTSeq--a Python framework to work with high-throughput sequencing data. *Bioinformatics (Oxford, England)* **31**, (2015).
20. M. M, W. PR, B. R, IntaRNA 2.0: Enhanced and Customizable Prediction of RNA-RNA Interactions. *Nucleic acids research* **45**, (2017).
21. A. Busch, A. S. Richter, R. Backofen, IntaRNA: efficient prediction of bacterial sRNA targets incorporating target site accessibility and seed regions. *Bioinformatics* **24**, 2849-2856 (2008).
22. M. B. Kery, M. Feldman, J. Livny, B. Tjaden, TargetRNA2: identifying targets of small regulatory RNAs in bacteria. *Nucleic Acids Res* **42**, W124-129 (2014).
