# Supplementary material for: A pathogen-specific sRNA influences enterohemorrhagic Escherichia coli fitness and virulence in part by direct interaction with the transcript encoding the ethanolamine utilization regulatory factor EutR
Source: Nucleic Acids Res. 2021 Sep 30;49(19):10988–1004. doi: 10.1093/nar/gkab863 (PMC8565329; doi:10.1093/nar/gkab863)
Supplement: gkab863_Supplemental_Files [file gkab863_supplemental_files.zip › Supplementaryfile1_legend.docx]

Supplementaryfile1

Processed MAPS and RNAseq data.
